# Recent Progress of Two-Dimensional Materials for Ultrafast Photonics

**DOI:** 10.3390/nano11071778

**Published:** 2021-07-08

**Authors:** Aojie Zhang, Zihao Wang, Hao Ouyang, Wenhao Lyu, Jingxuan Sun, Yuan Cheng, Bo Fu

**Affiliations:** 1 BUAA-CCMU Advanced Innovation Center for Big Data-Based Precision Medicine, School of Engineering Medicine, Beihang University, Beijing 100191, China; 18375484@buaa.edu.cn (A.Z.); 18374419@buaa.edu.cn (Z.W.); haooy@buaa.edu.cn (H.O.); 18375219@buaa.edu.cn (W.L.); sjxxjs@buaa.edu.cn (J.S.); zy2017317@buaa.edu.cn (Y.C.); 2 School of Instrumentation and Optoelectronic Engineering, Beihang University, Beijing 100191, China; 3 Key Laboratory of Big Data-Based Precision Medicine Ministry of Industry and Information Technology, Interdisciplinary Innovation Institute of Medicine and Engineering, Beihang University, Beijing 100191, China

**Keywords:** two-dimensional materials, fabrications, saturable absorbers, ultrafast lasers

## Abstract

Owing to their extraordinary physical and chemical properties, two-dimensional (2D) materials have aroused extensive attention and have been widely used in photonic and optoelectronic devices, catalytic reactions, and biomedicine. In particular, 2D materials possess a unique bandgap structure and nonlinear optical properties, which can be used as saturable absorbers in ultrafast lasers. Here, we mainly review the top-down and bottom-up methods for preparing 2D materials, such as graphene, topological insulators, transition metal dichalcogenides, black phosphorus, and MXenes. Then, we focus on the ultrafast applications of 2D materials at the typical operating wavelengths of 1, 1.5, 2, and 3 μm. The key parameters and output performance of ultrafast pulsed lasers based on 2D materials are discussed. Furthermore, an outlook regarding the fabrication methods and the development of 2D materials in ultrafast photonics is also presented.

## 1. Introduction

Nanomaterials are ultrathin materials with at least one-dimensional size in nanometers in three-dimensional space [1,2]. Nanomaterials have unique quantum natures, which make their properties different from general materials in the fields of force, heat, light, electricity, and magnetism [3,4,5,6,7,8,9]. In accordance with the structure morphology, nanomaterials can be divided into zero, one, two, and three dimensions. As a kind of nanomaterial, two-dimensional (2D) materials are composed of single or several atomic layers. Strong covalent or ionic bond forces in layers and weak van der Waals forces between layers endow 2D materials with fascinating electrical and optical properties, including the quantum Hall effect, high carrier mobility, and nonlinear optical properties [10,11].

In particular, 2D materials have attracted much attention in the field of ultrafast photonics due to their excellent saturable absorption properties [12]. Ultrafast lasers are the key development direction of ultrafast photonics, which have been widely used in various fields owing to their outstanding advantages, such as short pulse duration, high peak power, high repetition frequency, and wide spectral range [13,14,15]. The origin of ultrafast lasers can be traced back to 1966. At that time, DeMaria et al. obtained the first picosecond laser pulse output based on a neodymium glass laser by using passive mode-locking technology, opening up a new world for the research of ultrafast pulsed lasers [16].

Q-switching and mode-locking are two typical methods to achieve ultrafast pulsed lasers, both of which can be achieved by active and passive modulation [17,18]. Saturable absorber (SA) is an important medium for passive operation and can be divided into artificial and real ones. Artificial SA refers to forming a similar SA through nonlinear optical effects, such as nonlinear polarization rotator (NPR) and nonlinear optic loop mirror (NOLM) [19,20,21].

The first real SA is a semiconductor saturable absorber mirror (SESAM), which can realize mode-locking in different bands through parameter design with stable operation and low loss [22,23]. However, SESAM has a narrow working band, low damage threshold, complex preparation process, and high cost [24]. In the past few decades, nanomaterials have shown potential to be excellent SAs. Carbon nanotubes (CNTs) are a kind of one-dimensional materials with simple preparation and high cost-effectiveness that also have the key advantage of a high damage threshold [25,26,27]. However, the development of CNTs is restricted by non-saturable loss [28].

Three-dimensional (3D) materials, such as Dirac semimetal cadmium arsenide (Cd${}_{3}$As${}_{2}$), metal nanospheres, and nanoscale charcoal powder, can also be used as SAs. However, most 3D materials do not possess the saturable absorption property, and the large insertion loss limits further applications in ultrafast pulsed lasers [29,30,31,32,33,34,35,36,37]. Compared with artificial SAs, CNTs, and 3D materials, 2D materials are extensively used in ultrafast pulsed lasers as superior SAs owing to the excellent saturable absorption properties, tunable modulation depths, and broadband responses. Moreover, 2D materials possess various preparation methods and easy integration with laser cavities, as well as simplifying the light path of laser systems, which make them promising candidates for further applications in ultrafast lasers.

In 2004, Novoselov et al. used tape to repeatedly peel the bulk graphite according to the mechanical exfoliation (ME) method and acquired the stable single-layer graphene, which opened the door to the world of 2D materials [38]. Graphene, with its unique zero-band gap structure and excellent photoelectric properties [39,40,41], has rapidly become a research hotspot in the fields of optics, electricity, and biology. In 2009, Bao et al. embedded graphene as a SA in a fiber laser resonator for the first time, and then obtained a picosecond mode-locked pulse output [41]. Since then, 2D materials have gradually become a reliable choice of SAs for ultrafast pulse lasers due to their unique low dimensional physical characteristics, as well as the advantages of a wide working band, controllable modulation depth, and ultrafast relaxation time [42,43].

Thus far, the most widely studied 2D materials in the field of pulsed laser applications are graphene [40,41,44], topological insulators (TIs) [45,46], transition metal disulfides (TMDs) [47,48], and black phosphorus (BP) [49,50]. In addition, a growing number of novel 2D materials have been added to the 2D family, such as MXenes [51], bismuthine [52], and antimonene [53]. The above 2D materials have been widely explored for ultrafast laser applications from the visible to mid-infrared (mid-IR) wavelengths [54,55,56]. Various gain media have been combined with 2D SAs to achieve 1, 1.5, 2, and 3 μm, as well as other output wavelength pulsed lasers [57,58,59,60]. Methods for preparing 2D crystals have also been expanded, such as chemical vapor deposition (CVD), liquid phase exfoliation (LPE), and pulsed laser deposition (PLD) [61,62].

Here, we introduce a variety of methods for preparing 2D materials, and then the applications of typical 2D materials as SAs in ultrafast pulsed lasers are also summarized. Finally, we discuss the state of the art and challenges, as well as the future development of 2D materials in the Perspectives section.

## 2. Synthesis

**Figure 1:**
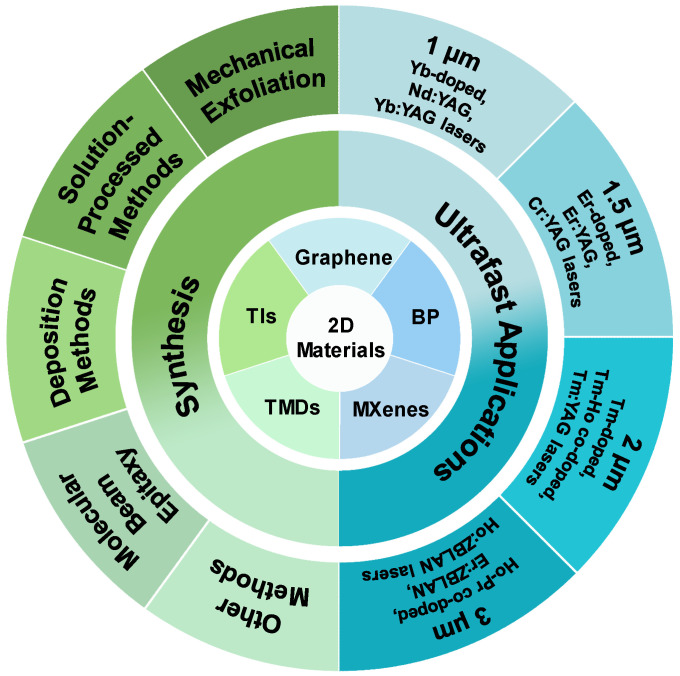
Synthesis and ultrafast applications of 2D materials.

In the past decade, 2D materials have attracted wide attention in applications of ultrafast lasers due to their excellent optical and electrical properties [43,63]. In this paper, we focus on the synthesis methods and the applications of widely studied 2D materials in common wavelength of ultrafast lasers (Figure 1). The synthesis of 2D materials is generally divided into top-down and bottom-up methods. In top-down methods, bulk materials are usually exfoliated to obtain 2D materials by physical or chemical methods to overcome the van der Waals force between layers.

**Figure 2:**
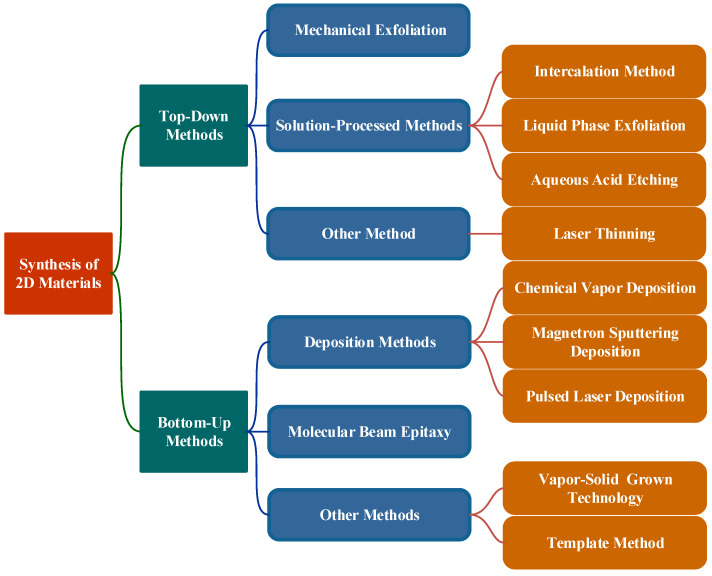
The synthesis of 2D materials.

As for bottom-up methods, 2D materials are usually obtained on the substrate by deposition or growth. As shown in Figure 2, we selected the widely used methods, including ME, solution-progressing methods, deposition methods, molecular beam epitaxy (MBE), and other methods. The principles, preparation processes, characteristics, and applications of these methods are also discussed.

### 2.1. Top-Down Methods

#### 2.1.1. Mechanical Exfoliation

The ME can separate single- and few-layer materials from bulk materials by overcoming the van der Waals force. Layered materials prepared by ME can be deposited on quartz substrates, such as silicon/silica (Si/SiO${}_{2}$), and cleaned by acetone, methanol, or isopropyl alcohol (IPA) to remove the scotch tape residue [64]. This method has the advantages of being simple, fast, and low cost, and the prepared layered materials have fewer defects and high integrity; therefore, ME is suitable for basic scientific research in laboratory. The ME method based on tape is the most widely used method.

**Figure 3:**
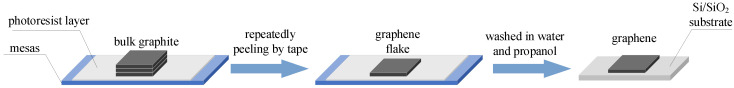
The preparation of a 2D material based on the ME method.

In 2004, Novoselov et al. used scotch tape to repeatedly peel the graphite sheet on mesas attached to the photoresist layer and first obtained single- and few-layer graphene (Figure 3) [38]. Yin et al. peeled a single-layer molybdenum dioxide (MoS${}_{2}$) sheet from a bulk sample and captured it onto on a Si/SiO${}_{2}$ substrate by using cellophane tape-based exfoliation. The height of the single-layer MoS${}_{2}$ obtained was ∼0.8 nm and the phototransistor made of it had good photo-responsivity [65]. ME is also widely used in the preparation of many other 2D materials. In 2010, Desalegne et al. described a method for “graphene-inspired” exfoliation of crystalline bismuth telluride (Bi${}_{2}$Te${}_{3}$) films with a thickness of a few atoms [66].

In 2013, Li et al. prepared single-layer TMDs sheets, such as tungsten diselenide (WSe${}_{2}$), tantalum disulfide (TaS${}_{2}$), and TaSe${}_{2}$, by using tape-based ME and then using Raman microscopy to characterize them [67]. Then, in 2014, Liu et al. made single-layer BP of ∼0.85-nm step height by tape-based exfoliation [64]. Other topological materials, including Bi${}_{2}$Se${}_{3}$ and tellurium antimonide (Sb${}_{2}$Te${}_{3}$), can also be obtained by the “graphene-like” ME from the bulk crystals [68].

In 2020, Gkountaras et al. obtained MXenes by the ME method with adhesive tape, in which the prepared MXenes had fewer defects than when using the etching method. This result indicated that ME can peel off some materials with a strong interlayer force, which could broaden the applications of the ME method [69]. However, the efficiency of ME is very low because it requires repeated peeling to obtain a certain number of monolayer materials. Therefore, this method is suitable for scientific research in laboratory; however, it is difficult to realize for mass production.

#### 2.1.2. Solution-Processed Methods

The solution-processed method is one of the most common methods to prepare 2D materials. This method is low cost, and the produced materials are easy to integrate [70]. Different from ME, this is a reliable method for the mass production of layered 2D materials. There are two main methods to prepare 2D materials in solution, including intercalation and LPE methods.

In the intercalation method, an ion or a molecule is usually inserted into the material to destroy the interlayer force [71]. Lithium (Li)-ion intercalation is the most commonly used method, which uses the reaction of water and Li atoms to generate a suspension in deionized water. After filtration or centrifugation, single-layer materials can be obtained. In 1986, Joensen et al. first prepared single-layer MoS${}_{2}$ by Li-ion intercalation, in which MoS${}_{2}$ was immersed in a solution of n-butyl Li in hexane for 48 h to achieve intercalation. The intercalated materials were placed into water, and then the monolayer MoS${}_{2}$ was obtained by ultrasonic treatment and centrifugation after the gas was released [71].

In 1996, Yang et al. also prepared monolayer WS${}_{2}$ by the intercalation method. They soaked the WS${}_{2}$ powder in the solution of butyl Li in hexane to obtain Li${}_{x}$WS${}_{2}$ and controlled the temperature to ensure x > 1. Then, in order to obtain a WS${}_{2}$ suspension, Li${}_{x}$WS${}_{2}$ was placed into distilled water. After washing and centrifugation repeatedly, single-layer WS${}_{2}$ was obtained [72]. However, the ion intercalation method is time-consuming and sensitive to the environment, and the prepared material tends to aggregate during deposition [73]. On the basis of the ion intercalation method, some improved methods have been proposed.

In 2012, Ren et al. proposed the hydrothermal intercalation/exfoliation method, which can synthesize Bi${}_{2}$Te${}_{3}$ nanosheets on a large scale conveniently. They placed Bi${}_{2}$Te${}_{3}$ bulk crystals into a teflon-lined autoclave (30 mL) filled with an ethylene glycol solution of Li hydroxide at 200 °C heated for 24 h to finish Li-ion intercalation. Then, a Bi${}_{2}$Te${}_{3}$ colloidal suspension was prepared in deionized water, and the nanosheets were obtained after filtration and drying [74].

**Figure 4:**
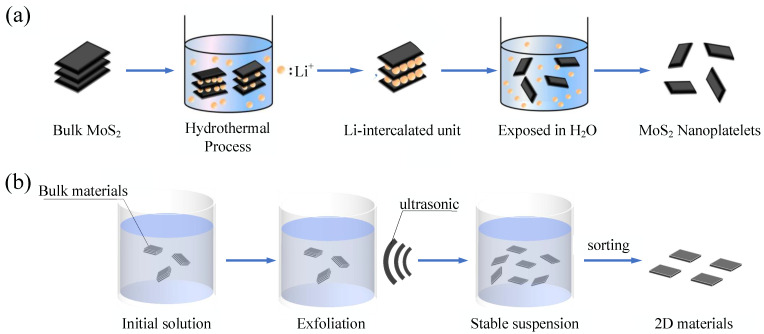
The preparation of 2D materials based on solution-processed methods. (**a**) Schematic of MoS${}_{2}$ fabricated by hydrothermal intercalation/exfoliation. Adapted from [47]. (**b**) Schematic of 2D materials fabricated by liquid phase exfoliation (LPE).

In 2014, Zhang et al. completed the intercalation of Li ion by the hydrothermal method and obtained a MoS${}_{2}$ nanoparticle membrane. As shown in Figure 4a, they placed bulk MoS${}_{2}$ in an autoclave filled with Li ions and heated it at 200 °C for 72 h to achieve Li-ion intercalation. Then, as with other Li-ion intercalation methods, after placing the intercalated materials into water, a MoS${}_{2}$ nanoparticle membrane was obtained by filtration [47]. The hydrothermal intercalation/exfoliation method was also used to prepare Bi${}_{2}$Se${}_{3}$ [75,76]. Apart from Li-ion intercalation and hydrothermal intercalation/exfoliation, electrochemical intercalation that is slow but can precisely control the number of layers is also a commonly used method [77,78].

Different from the ion intercalation that often occurs in chemical reactions, LPE is a kind of physical method with a simple process and easy realization. In general, the van der Waals force between the layers of materials is destroyed by using high-intensity ultrasound, and then 2D materials are selected by centrifugation to prepare layered 2D nanomaterials. In 2008, Hernandez et al. demonstrated, for the first time, that LPE can be used to prepare a large number of graphene nanosheets [61]. Subsequently, other 2D materials, such as BP [79,80,81,82], boron nitride (BN) [83,84], TMDs [83,84], and TIs [85,86,87,88,89], were also fabricated successfully by LPE.

LPE is generally divided into three main steps, including exfoliation, stabilization, and sorting (Figure 4b). Exfoliation is the process of breaking the force between layers of materials and generating a single or multi-layer structure. The most commonly used method of exfoliation in liquid is ultrasonication, which has the advantages of being simple, low cost, and of immediate use [90,91]. High-power sonic probes [92,93], sonic baths [61,94,95,96], and tip ultrasonicators [92,93] are usually used to produce ultrasonication.

There are also other methods for exfoliation, such as ball milling [97,98,99] and shear exfoliation [100]. After exfoliation, the 2D material needs to be stabilized in the liquid phase. In order to avoid material reaggregation and to reduce the net energy cost of exfoliation, suitable stabilizers are needed, of which the solvent, surfactant, and polymer are the three main ones. N-methyl-2-pyrrolidone and dimethylformamide are two common organic solvents used in the LPE method. However, due to their high boiling point and toxicity, people have also attempted to use IPA with a low boiling point [80,81,101], co-solvent, such as water/ethanol [102,103], or water/isopropanol [103] as a substitute.

Sodium dodecylbenzene sulfonate and sodium cholate can be used as surfactants to prevent reaggregation of materials in the liquid by electrostatic or spatial repulsion [84,96,104,105]. Moreover, simple kitchen soap [106] and aromatic molecules [107] have also been shown to be used as surfactants in the preparation of 2D materials. Sometimes polymers, such as proteins and copolymers with hydrophobic centers and hydrophilic chains, can be used as substitutes for surfactants, which are adsorbed on the surface of nanosheets, increasing the repulsive force between the nanosheets, thus, maintaining the stability of 2D materials [108,109,110]. Reserach proved that these polymers can be used in graphene [108] and some TMDs [109,111].

After stabilization, 2D materials with the appropriate transverse size and thickness need to be sorted from the stripped dispersions. The commonly used method is ultracentrifugation, which can be realized by sedimentation based-separation (SBS) and density gradient ultracentrifugation (DGU) [112]. SBS separates different dispersions by their different sedimentation rates and the larger dispersions generally settle faster than the smaller ones [112]. At present, using SBS can sort many 2D materials [83,84,113]; however, it can only separate dispersions with a small transverse size. For a dispersion with a large settlement coefficient, it is not effective.

Compared with SBS, DGU can achieve more accurate control of layer number, in which the dispersed nanomaterials are ultracentrifuged in the preformed density gradient medium (DGM) [114]. In this process, dispersions move along the tube under the action of centrifugal force until they reach the point where their buoyancy density equals the buoyancy density of the surrounding DGM, and therefore materials with different layers are in different gradient positions [115]. However, DGU is time-consuming and low yield; thus, researchers can choose the appropriate method according to the actual situation between SBS and DGU.

In general, LPE has the advantages of scalability, room temperature treatment, and high yield; however, the problems of difficulty to accurately control the size and thickness of the exfoliated layer materials also limit its applications.

Aqueous acid etching (AAE) is the most commonly used and efficient method to prepare MXenes, which are exfoliated from MAX phases. The molecular formula of the MAX phase is M${}_{n+1}$AX${}_{n}$, where M is early transition metal elements, A represents group IIIA or IVA elements, and X represents C and/or N. In AAE, M-A bonds are destroyed in the MAX phase by chemical etching the atomic layer. Hydrogen fluoride (HF) is the main etchant to prepare MXenes with low cost and mature technology. In 2011, Naguib et al. used HF to etch titanium aluminum carbide (Ti${}_{3}$AlC${}_{2}$) and obtained the first MXene (Ti${}_{3}$C${}_{2}$T${}_{x}$) [116].

Later, HF was gradually used in more MAX phases to prepare MXenes [117,118,119,120,121]. However, when manufacturing MXenes with HF, the parameters, such as the HF concentration and etching time, need to be strictly controlled, and HF is highly corrosive and harmful to human body. As a result, other etching solutions for the acid etching of MXenes have been explored. For example, Halim et al. prepared Ti${}_{3}$C${}_{2}$ by selectively etching Al in Ti${}_{3}$AlC${}_{2}$ thin films with ammonium hydrogen fluoride (NH${}_{4}$HF${}_{2}$) aqueous solution [122]. Moreover, lithium fluoride (LiF) and hydrogen chloride (HCl) mixed solution has also been proven to be able to prepare MXenes, such as Ti${}_{3}$C${}_{2}$ [123], Ti${}_{2}$C [124,125], and Ti${}_{3}$CN [126].

#### 2.1.3. Other Methods

Laser thinning is a top-down method to obtain 2D materials by laser thinning multi-layer materials on the substrate. It can realize the precise control of the number of layers and patterns and has high repeatability, indicating the potential for preparing 2D materials [127,128,129]. In the laser thinning method, multi-layer thin films are deposited on the substrate by ME [127,128,129,130] or chemical deposition [131,132,133], then laser scanning is used to refine the multi-layer into a single layer.

**Figure 5:**
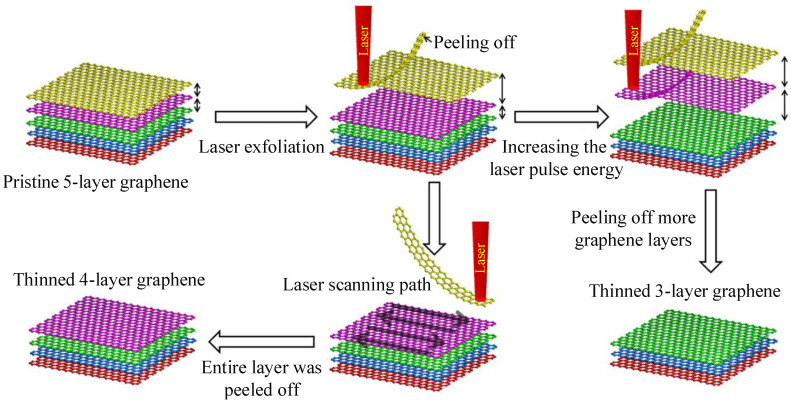
The preparation of 2D material based on the laser thinning method. Adapted from [133].

The process of laser thinning is shown in Figure 5. In 2011, Han et al. thinned the CVD-grown graphene film by laser irradiation, realized the preparation of single-layer graphene, and investigated the key parameters of the thinning effect [131]. In 2012, Castellanos-Gomez et al. deposited multi-layer MoS${}_{2}$ films on a Si/SiO${}_{2}$ substrate by ME, which was thinned by a high-power scanning laser. Finally, they reduced the multi-layer MoS${}_{2}$ into a single-layer 2D crystal at a rate of 8 μm${}^{2}$/min by laser thinning [127]. Later, in order to make the 2D materials prepared by laser thinning better used in electronic devices, precise controls of layer number and pattern have been widely studied. In 2015, Lin et al. thinned multi-layer graphene by using a picosecond laser, which obtained the required number of layers [133].

In the same year, Li et al. realized the thinning of graphene to a specific thickness by using femtosecond laser grating scanning [132]. In 2017, Hu et al. also achieved layer-by-layer precision patterning in the preparation of MoS${}_{2}$ films by vertical and lateral control, where they also quantitatively designed the laser power and exposure time for accurate thinning and patterning [129]. Up to now, laser thinning has been mainly used in the preparation of graphene and MoS${}_{2}$; however, as a method of high precision and repeatability, laser thinning also has potential in the preparation of other 2D materials.

### 2.2. Bottom-Up Methods

#### 2.2.1. Deposition Methods

**Figure 6:**
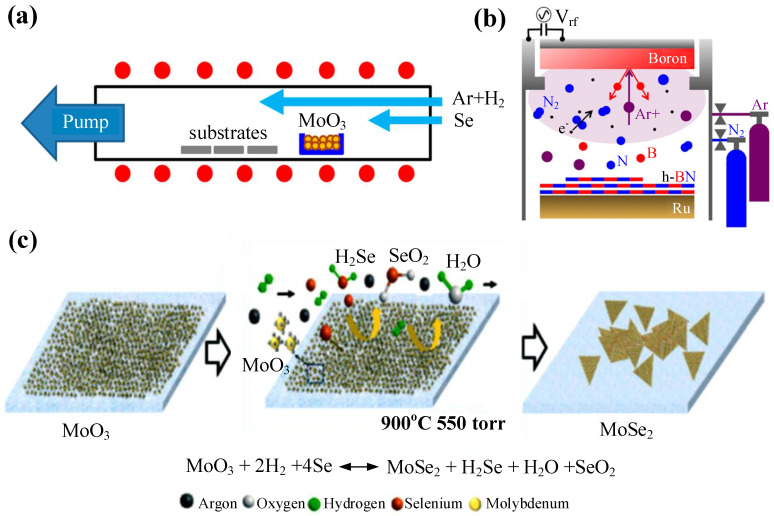
The preparation of 2D materials based on deposition methods. (**a**) Schematic of layered MoSe${}_{2}$ grown by CVD. Adapted with permission from reference [137]. Copyright 2010 American Chemical Society. (**b**) Schematic of BN films grown by MSD. Adapted with permission from [140]. Copyright 2012 American Chemical Society. (**c**) Schematic of MoSe${}_{2}$ monolayer grown by PLD and selenidation techniques. Adapted with permission from [141]. Copyright 2016 The Royal Society of Chemistry.

CVD is a process where a gaseous substance reacts in the gas phase or on the substrate surface to produce 2D material films. LPE and ME can only produce 2D materials with a small size, while CVD can produce large-area and uniform materials [134,135]. In 2008, Yu et al. used CVD to synthesize graphene for the first time, as well as investigating the effects of cooling rate and other growth conditions on the thickness and defect number of graphene [136]. A year later, Li et al. grew uniform graphene on a metal surface by CVD, in which they used a mixture of methane and hydrogen to grow graphene on 25-mm copper foil [134]. In 2014, Chang et al. prepared crystalline monolayer MoSe${}_{2}$ by CVD with MoO${}_{3}$ and Se powder. As shown in Figure 6a, argon and hydrogen bring MoO${}_{3}$ and Se vapor onto sapphire substrate, then the two react to grow MoSe${}_{2}$ on the substrate [137]. In 2015, Xu et al. first prepared large-area and high-quality 2D ultrathin Mo${}_{2}$C crystals on copper foil using the CVD method, and it was easy for them to adjust the size and thickness of 2D α-Mo${}_{2}$C crystal by changing the experimental regulation [138]. One advantage of CVD is that, for SA, the modulation depth can be increased by controlling the number of layers. In 2018, Liu et al. prepared WSe${}_{2}$ thin films by CVD and successfully obtained different modulation depths by controlling the thickness of WSe${}_{2}$ thin films [139].

Another advantage of CVD is the good versatility can be used to prepare a variety of 2D materials. In 2010, Shi et al. obtained hexagonal boron nitride (h-BN) films with a thickness between 5 and 50 μm and transverse size up to 20 μm on nickel (Ni) films by CVD under atmospheric pressure [142]. In 2014, Zhang et al. obtained uniform and high-quality Bi${}_{2}$Se${}_{3}$ thin films in a selenium-enriched environment by CVD without a catalyst, which reduced the preparation cost, and the effects of certain parameters on the growth process were discussed in detail [143]. In 2020, Gu et al. prepared high-quality 2D palladium selenide (PdSe${}_{2}$) crystals based on atmospheric pressure CVD method and studied the growth process and morphology of PdSe${}_{2}$ crystals by experimentation and theoretical calculations [144].

In order to prepare high-quality 2D materials by CVD, it is also necessary to control the parameters that have great influence on the size and number of layers of the final 2D materials [145]. For example, in 2020, Tang et al. proposed a vertical CVD method to achieve uniform, high-quality, and reproducible growth of single-layer TMDs by controlling the precursor concentration, gas flow, and temperature [146]. In general, the superiorities of CVD are that it can prepare large-area and uniform 2D materials, the yield is high, and the number of layers is easy to control. However, there are certain drawbacks, such as the complex preparation process [147], high cost [148], and certain risk [149], which need to be considered in the actual situation.

During the preparation process of magnetron sputtering deposition (MSD), the target material is bombarded by the ions, which are accelerated in a strong magnetic and electric field. Then, the splashed material reacts with the material in the gas to obtain 2D materials on the substrate. MSD has a high deposition rate and low growth temperature, in which the deposition rate can be controlled by adjusting the effect of the radio-frequency (RF) power [150]. Li et al. prepared polycrystalline indium selenide (In${}_{2}$Se${}_{3}$) thin films by MSD at 270 and 380 °C, as well as found a linear relationship between the deposition rate and effect of RF power where the fastest speed could reach 121.7 nm/min.

In 2013, Sutter et al. obtained high-quality and controllable thickness BN films by designing different schemes MSD of B in N${}_{2}$/argon (Ar). As shown in Figure 6b, the collision of high-energy Ar ions with a solid B target resultd in the evaporation of B. Then, h-BN was obtained by the combination of evaporated B and dissociated N${}_{2}$ on the substrate [140]. In 2015, Ling et al. prepared MoS${}_{2}$ by MSD, and the film thickness could be controlled to 0.75 nm [151]. In the same year Tao et al. demonstrated a MSD method for synthesizing uniform MoS${}_{2}$ with controllable number of layers in a large area [152].

In 2016, Zhang et al. deposited Bi${}_{2}$Se${}_{3}$ thin films with good crystalline quality on Si within 10 min by using Bi${}_{2}$Se${}_{3}$ alloy with purity of 99.999% as target in MSD and discussed the resistivity characteristics of the films [153]. MSD has the advantages of simple and easy control, as well as low cost and high speed; however, it also has the problems of high energy consumption and poor material quality [28,152].

In 1987, Dijkkamp et al. successfully prepared yttrium–barium–copper–oxygen superconductor thin films by pulsed excimer laser evaporation of single bulk target in a vacuum for the first time [154]. Since then, PLD technology has gradually emerged. PLD is a bottom-up physical vapor deposition technology, which uses high-energy pulsed laser to impact the target in a high vacuum and then produce a plasma plume. The plume reaches the substrate and crystallizes into the target material [155]. A PLD system generally consists of an excimer laser, optical system, and growth chamber equipped with a vacuum pump and gas path [155].

Thus far, PLD has been widely used in the synthesis of 2D materials. In 2011, Wang et al. achieved the synthesis of large-area graphene (1 × 1 cm${}^{2}$) on Ni thin films by PLD [156]. Subsequently, Onsoe et al. synthesized high-quality Bi${}_{2}$Se${}_{3}$ atom planar topological insulator epitaxial films with controllable thickness in the range of 6–120 nm using the same method [157]. In 2014, Lee et al. also obtained high-quality Bi${}_{2}$Se${}_{3}$ thin films on a Al${}_{2}$O${}_{3}$ substrate with low repetition rate and deposition temperature in a multi-target PLD cavity [158]. In 2015, Yang et al. grew ultrathin BP films on graphene/copper or SiO${}_{2}$/Si substrates with bulk BP crystal as target, in which uniform film growth was achieved by rotating the substrate and target [159].

In 2016, Farman et al. first deposited MoO${}_{3}$ thin films by PLD, and then obtained MoSe${}_{2}$ thin films by selenization in a two zone hot-wall furnace, as shown in Figure 6c [141]. Moreover, PLD is also a high degree of freedom technology, and thus many requirements can be achieved by adjusting the parameters, such as the substrate cooling rate, gas atmosphere, gas pressure, laser frequency, and laser energy. PLD has the advantages of simple operation, high efficiency, low pollution, good versatility, and substrate compatibility [28]. However, the research of PLD has just begun. PLD is expected to produce a variety of 2D materials in a large area and realize industrialization in the future.

#### 2.2.2. Molecular Beam Epitaxy

MBE is a layer by layer deposition technique, which can epitaxially grow 2D thin films on substrates. It is noted that MBE is a clean, safe, expandable, and highly controllable method and has the advantages of low growth temperature and low impurity caused by a high vacuum [160,161]. In 2014, Zhang et al. grew Sb${}_{2}$Te${}_{3}$ thin films with a thickness of 28–121 nm on Si substrates at 280 °C by MBE [162]. Zhu et al. successfully prepared 2D stanine based on Bi${}_{2}$Te${}_{3}$ substrates, in which 40-nm Bi${}_{2}$Te${}_{3}$ thin films were first grown on Si wafers by MBE [163].

**Figure 7:**
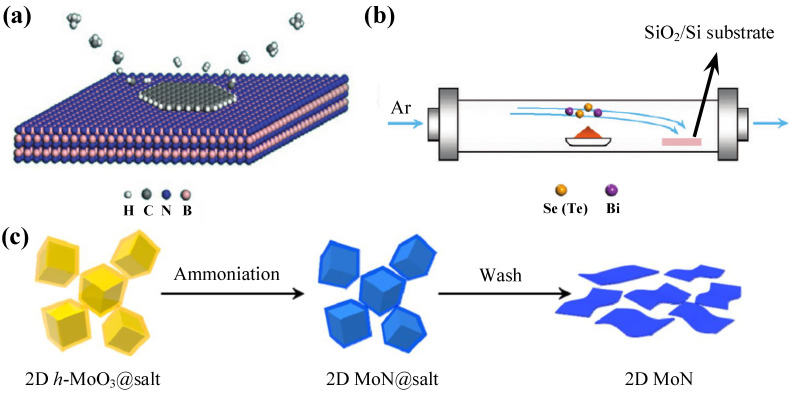
The preparation of 2D materials based on MBE, VS, and template methods. (**a**) Schematic of graphene fabricated by epitaxial growth. Adapted with permission from [164]. Copyright 2013 Macmillan Publishers Limited. (**b**) Schematic of Bi${}_{2}$Se${}_{3}$ and Bi${}_{2}$Te${}_{3}$ fabricated by VS growth. Adapted with permission from [165]. Copyright 2010 American Chemical Society. (**c**) Schematic of 2D MoN fabricated by template method. Adapted with permission from [166]. Copyright 2017 American Chemical Society.

In later research, plasma was proven to play an auxiliary role in MBE. Therefore, Mishra et al. grew Gallium nitride (GaN) on a monolayer-MoS${}_{2}$/c-sapphire substrate by plasma-assisted MBE [161]. Yang et al. also successfully obtained large-area graphene single crystals on h-BN with fixed stacking orientation by plasma assisted deposition for the first time. As shown in Figure 7a, on the h-BN crystal obtained by ME, various free radicals decomposed from methane epitaxially grew graphene along the edge, thus, expanding the area of graphene [164].

Currently, many 2D materials can be prepared by MBE, such as MoTe${}_{2}$ [167], WTe${}_{2}$ [168], Bi${}_{2}$Te${}_{3}$ [169], Bi${}_{2}$Se${}_{3}$ [170], MoS${}_{2}$ [161], MoSe${}_{2}$ [171], and WSe${}_{2}$ [172]. However, MBE also faces some challenges, such as high cost caused by the complex ultra-high vacuum system, slow speed caused by precise control, as well as limitations on the available materials and substrates caused by strict lattice matching constraints [28].

#### 2.2.3. Other Methods

In addition to the general methods described above, there are also some specific methods to prepare a certain kind of 2D materials. In 2010, Kong et al. developed a catalyst free vapor-solid (VS) growth technology. They placed Bi${}_{2}$Se${}_{3}$ or Bi${}_{2}$Te${}_{3}$ powder of 99.999% purity as raw materials in a horizontal tube furnace, then heated and deposited them on SiO${}_{2}$/Si substrate by Ar gas transport (Figure 7b). Using this method, they obtained Bi${}_{2}$Se${}_{3}$ and Bi${}_{2}$Te${}_{3}$ ultrathin nanoplates with a thickness of 3 nm [165].

In 2019, Khan et al. obtained 2D Bi${}_{2}$O${}_{2}$Se single crystals at the millimeter scale by VS growth technology in atmospheric pressure and proved that this material had good crystalline quality, chemical uniformity, and stoichiometry [173]. The template method has also received wide attention in recent years. In 2016, Xiao et al. used salt microcrystals as substrates/templates to guide the growth of oxides at elevated temperature, which can synthesize transition metal oxides, such as hexagonal-MoO${}_{3}$, MoO${}_{2}$, MnO, and WO${}_{3}$ [174].

In 2017, Xiao et al. also obtained 2D nitrides, such as MoN, W${}_{2}$N, and V${}_{2}$N, by ammoniating the oxide, as well as confirming the metal properties of 2D MoN and its excellent rate performance in sulfuric acid electrolytes. As shown in Figure 7c, they first obtained 2D hexagonal MoO${}_{3}$-coated NaCl (2D h-MoO${}_{3}$@NaCl) through the salt template method, and then the powder was annealed at 650 °C for 5 h in a mixture of NH${}_{3}$ (5%) and Ar to obtain 2D MoN@NaCl. Finally, 2D MoN was obtained after deionized water cleaning. By changing the parameters and precursors, other 2D metal nitrides or carbides can also be synthesized by the template method [166].

To sum up, there are a variety of methods to prepare 2D materials, each of which has its own characteristic and application scenarios. In top-down methods, ME is often used in the scientific research of laboratories due to its high integrity of materials. However, there is a problem meaning that it cannot be used in mass production. Solution-processed methods, especially LPE, provide the method for large-scale preparation of 2D materials owing to the low cost and simple operation. As for the accurate and reproducible preparation of 2D materials, we introduce the laser thinning method. Although this method is mainly used to prepare graphene and MoS${}_{2}$, its potential and possibility are also worth exploring.

In terms of bottom-up methods, CVD is a common preparation method, which can produce uniform and large-area materials with high yield, as well as control the number of layers and size by adjusting the parameters. In view of the high cost and complex operation of CVD, MSD with low cost and simple operation can be used to replace CVD in some application scenarios, where the quality requirements of materials are not particularly high. If the requirements for thickness and shape are high, PLD with a high degree of freedom is a better choice. MBE as a mature method, and its vacuum and pure characteristics make it suitable for the preparation of high-purity 2D materials in the laboratory for research. In addition to general top-down and bottom-up methods, AAE, VS growth technology, and the template method, which are used for the synthesis of specific categories of 2D materials are also being explored.

## 3. Applications of 2D Materials in Ultrafast Lasers

Ultrafast lasers are the key component of ultrafast photonics, which have come into practice in various fields, such as micromachining [175], communication [176], medical procedures [177,178], gas detection [179], and remote sensing [180]. With the advantages of stability, compactness, and easy implementation, mode-locking and Q-switching are two notable techniques to achieve ultrafast pulsed lasers, where SAs perform crucial roles in many types of ultrafast lasers, such as fiber, solid-state, and waveguide lasers [17,18,49,181,182,183].

Over the last decade, 2D materials have been extensively investigated and applied as reliable SAs in ultrafast lasers at series of wavelengths due to their innate nonlinear saturable absorption properties, broadband operation, and ultrafast relaxation time [43,184,185]. Here, we summarize and discuss diverse types of ultrafast lasers operated at the commonly used wavelengths (1, 1.5, 2, and 3 μm), which are enabled by the most widely studied 2D materials, including graphene, TIs, TMDs, BP, MXenes, and their heterostructures.

### 3.1. Graphene

As the pioneer of 2D materials, graphene came to the first use as SA in 1.5-μm region due to the telecommunication boom. In 2009, Hasan et al. first reported on an ultrafast laser mode-locked by a solution-processed graphene-polymer at 1557 nm with a pulse duration of ∼800 fs [40]. Almost at the same time, Bao et al. demonstrated the use of graphene SA as mode-locker in an erbium-doped fiber laser (EDFL) at 1565 nm, in which a graphene film was synthesized by CVD and coated on the core of a fiber [41]. Subsequently, Zhang et al. employed a graphene-polymer and atomic layer graphene as SAs in an EDFL to achieve large-energy mode-locked pulses with single pulse energies of 3 and 7.3 nJ, respectively [186].

**Figure 8:**
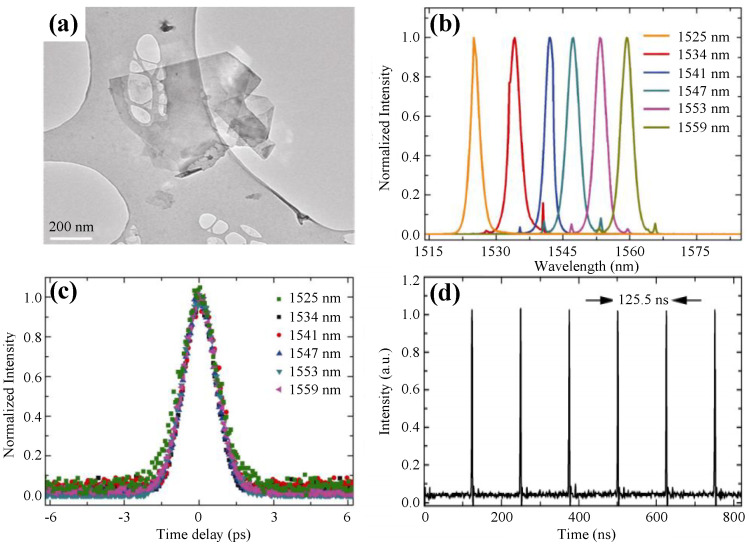
The experimental results of mode-locked EDFL enabled by graphene SA. (**a**) TEM image of a folded graphene flake. (**b**) Output spectra. (**c**) Autocorrelation traces. (**d**) Output pulse train. (**a**–**d**) adaped from [188].

Since this pioneering work, the applications of 2D materials including graphene in ultrafast lasers started flourishing [187]. In 2010, Sun et al. proposed a broadband tunable ultrafast laser mode-locked by graphene at the central wavelengths ranging from 1525 to 1559 nm with a pulse width about 1 ps near transform limitation [188]. Such wavelength-tunable pulsed lasers enjoy great potential in various applications, such as spectroscopy and sensing [189]. Figure 8a shows the transmission electron microscope (TEM) image of the folded graphene flake. The output spectra, corresponding autocorrelation traces, and output pulse train are shown in Figure 8b–d, respectively.

After abundant work on mode-locked lasers, graphene started to be applied in Q-switched lasers to gain pulses with high pulse energy [190]. Luo et al. first exploited graphene as SA to obtain Q-switched pulses in EDFL, which achieved dual-wavelength pulses at 1566.17 and 1566.35 nm with a pulse width of 3.7 μs and pulse energy up to 16.7 nJ [39]. In the next year, a graphene-based Q-switched fiber laser with a tunable broadband between 1522 and 1555 nm was demonstrated by Popa et al., in which a pulse width of 2 μm and pulse energy of 40 nJ were obtained [191]. Such a broadband laser could be applied as the light source for metrology [192], biomedical diagnostics [193], and environmental sensing [194].

In 2012, Gao et al. reported on the first use of graphene SA in a Q-switched Er-doped yttrium aluminum garnet (Er:YAG) laser at 1645 nm with a repetition rate of 35.6 kHz and maximum output power of 251 mW [195]. Soon after that, Sobon et al. realized a passive harmonic mode-locked EDFL with atomic multilayer graphene SA, where a high repetition rate of 2.22 GHz was achieved at the 21st harmonic [196]. In 2013, Fu et al. also reported on a 32nd harmonic mode-locked laser with excellent stability, in which the supermode suppression was up to 50 dB and the signal-to-noise ratio (SNR) was better than 67 dB [44]. Cafiso et al. deployed monolayer graphene in a Chromium-doped YAG (Cr:YAG) laser, obtaining stable mode-locked pulses with a short pulse duration of 91 fs [197].

In 2015, Sotor et al. integrated a CVD-grown graphene-polymer composite (60-layer) into a dispersion-managed fiber laser and obtained a stretched mode-locked pulse of 88 fs in 1.5 μm [198]. The study also validated that the modulation depth of multilayer graphene was proportional to the number of its layers, which can reach up to the 10% level. In 2019, Fu et al. demonstrated the bound states of solitons and harmonic mode-locking from a fiber laser based on graphene [199]. The laser could produce 26th harmonic stable pulses with a pulse duration of 720 fs and an SNR of 65 dB at the repetition rate of 409.6 MHz, which promoted the applications of graphene-based lasers, including spectroscopy and nonlinear imaging.

The research of graphene-based ultrafast laser in 1 μm was carried out simultaneously along with that in 1.5 μm. In 2010, Yu et al. reported on the first Q-switched ultrafast laser by employing graphene epitaxially grown on silicon carbide (SiC) in a neodymium-doped YAG (Nd:YAG) crystal laser centered at 1064 nm, obtaining a maximum pulse energy of 159.2 nJ [200]. Subsequently, Zhao et al. proposed the first mode-locked ytterbium-doped fiber laser (YDFL) based on CVD-grown graphene film with a dissipative soliton pulse of 580 ps [201]. In 2011, a graphene-based Q-switched YDFL was demonstrated by Liu et al. [202]. In the experiment, the graphene polyvinyl-alcohol (PVA) composite was deposited on a broadband reflective mirror, and the laser generated stable pulses with a pulse width of 70 ns at maximum output power of 12 mW.

In 2011, Cho et al. proposed the first graphene-based solid-state laser with a tunable wavelength from 1.22 to 1.25 μm, where stable 94-fs pulses with an SNR of 62.2 dB were achieved at a repetition rate of 75 MHz [203]. In 2014, Zhao et al. realized a rectangular pulsed fiber laser mode-locked by microfiber-based graphene, the operating wavelength of which could be set to 1061.8 and 1068.8 nm by tuning the bandpass filter or rotating the polarization controllers [204].

In 2011, Wang et al. first used graphene as SA in a Q-switched thulium-doped YAG (Tm:YAG) laser at the wavelength of 2-μm, achieving a pulse energy up to 1.74 μJ and a maximum average output power of 38 mW [205]. In 2012, Ma et al. reported on the first mode-locked laser enabled by graphene-polymer composite at 2018 nm with a pulse width of 729 fs, where Tm-doped calcium lithium niobium gallium garnet (Tm:CLNGG) crystal was used as gain media [206]. Later, Zhang et al. reported on a Tm-doped fiber laser (TDFL) mode-locked by graphene-polymer, achieving low-noise pulses with a pulse width of 3.6 ps and an SNR of ∼70 dB [207].

Liu et al. achieved the first Q-switched operation based on graphene SA in 2 μm, which possessed a pulse duration of 1.4 μs and a single pulse energy of 85 nJ [208]. These works laid the foundation for the applications of graphene SA in 2-μm region. In 2013, Fu et al. achieved a pulse energy up to 35 nJ in a graphene-based mode-locked Tm-Ho co-doped fiber laser (THDFL), which possessed a tunable waveband from 1897.69 to 1930.27 nm [209]. Jiang et al. proposed an ultrafast TDFL Q-switched by CVD-grown graphene SA with a maximum output power of 96 mW at the repetition rate of 202 kHz [210].

In 2014, Zhao et al. achieved Q-switched pulses in a graphene-based holmium-doped YAG (Ho:YAG) ceramic laser, obtaining a pulse energy up to 9.3 μJ [211]. Furthermore, Fu et al. employed graphene as SA in ytterbium (Yb)-, Er-, and Tm-Ho co-doped fiber lasers successively and all achieved passively mode-locking, which demonstrated the broadband operation property of graphene in wide operating wavelengths from 1 to 2 μm [55].

As for the mid-IR region, the first graphene mode-locked Cr:Zinc Selenide (Cr:ZnSe) laser centered around 2.5 μm was proposed by Cizmeciyan et al. in 2013 [212]. Soon after that, Tokita et al. reported on the first graphene Q-switched laser in 3 μm by incorporating a graphene SA mirror in an Er-doped ZBLAN fiber laser, obtaining a repetition rate of 59 kHz, a pulse width of 400 ns, and an average output power of 380 mW, respectively [213].

In 2015, Zhu et al. demonstrated a mode-locked fiber laser in 3-μm region for the first time based on graphene SA mirror [214]. According to the results, mode-locked pulses had a pulse width of 42 ps at a repetition rate of 25.4 MHz. Without stopping, the working wavelength of ultrafast mode-locked laser based on graphene kept extending, which reached up to 4.4 μm under Pushkin et al.’s work in 2020 [215].

Unfortunately, the application of graphene SA is limited due to its low absorption co-efficiency (2.3% per layer) and absence of bandgap [41,82]. However, the modulation depth of graphene increases with number of its layers, which enables the remarkable performance of graphene in broadband operations [41,70]. Additionally, other merits compared with traditional SA, such as the ultrafast recovery time, lower saturation intensity, and wavelength-independent saturable absorption, make graphene an outstanding SA that is widely used in mode-locked and Q-switched lasers [201,216]. It is the applications of graphene in ultrafast lasers that opened the curtain on the research of 2D materials in ultrafast photonics with excellent performance.

### 3.2. Topological Insulators

Due to the similarity to graphene in band structure [87,217,218], TIs, such as Bi${}_{2}$Te${}_{3}$, Bi${}_{2}$Se${}_{3}$, and Sb${}_{2}$Te${}_{3}$, have also been widely studied and applied in ultrafast photonics. In 2012, Bernard et al. made a preliminary exploration of the optical properties about Bi${}_{2}$Te${}_{3}$ and used it as SA to realize a mode-locked fiber laser in the 1.5-μm region [45]. Triggered by this work, Zhao et al. also demonstrated a mode-locked laser based on Bi${}_{2}$Te${}_{3}$ that was fabricated by the hydrothermal intercalation/exfoliation method [217].

**Figure 9:**
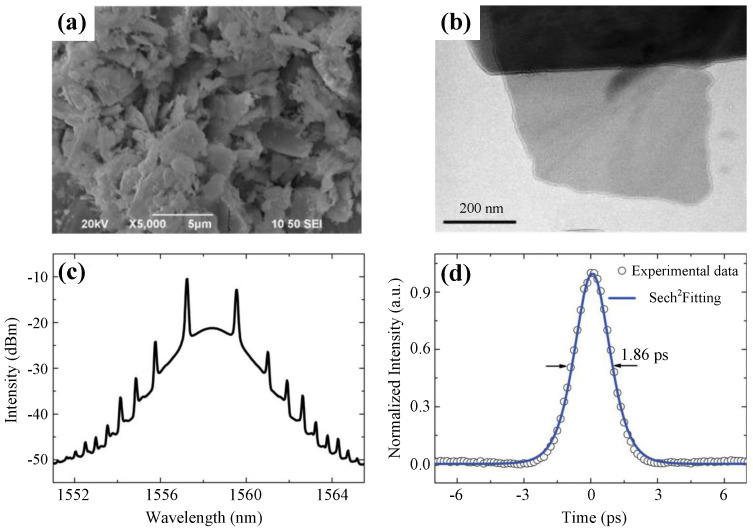
The experimental results of mode-locked EDFL enabled by Bi${}_{2}$Te${}_{3}$ SA. (**a**) SEM and (**b**) TEM images of Bi${}_{2}$Te${}_{3}$ nanosheets prepared by the hydrothermal intercalation/exfoliation method. (**c**) Soliton spectrum. (**d**) Autocorrelation trace. (**a**–**d**) adapted with permission from [217]. Copyright 2012 American Institute of Physics.

Figure 9c,d shows the spectrum centered at 1558.4 nm and the pulse width of 1.86 ps. Scanning electron microscopy (SEM) and TEM images of the as-prepared Bi${}_{2}$Te${}_{3}$ are shown in Figure 9a,b. Later, the saturable absorption property of Bi${}_{2}$Se${}_{3}$ was experimentally investigated by Zhao et al. from a mode-locked fiber laser with a tunable waveband ranging from 1557 to 1565 nm [87]. This pioneering work paved the path for applications of TIs in ultrafast photonics.

In 2013, Tang et al. obtained Q-switched operation from a Bi${}_{2}$Te${}_{3}$-induced Er:YAG ceramic laser at 1.645 μm with a maximum output power of 210 mW, which indicated that TIs SAs could be suitable candidates for high-power applications [46]. In the same year, Chen et al. achieved a dual-wavelength fiber laser at 1545.85 and 1565.84 nm, and a wavelength-tunable fiber laser ranging from 1510.9 to 1589.1 nm both Q-switched by Bi${}_{2}$Se${}_{3}$ successively, which confirmed the potential of TIs in broadband optical operation [86].

In 2014, Lee et al. demonstrated a femtosecond mode-locked fiber laser at the central wavelength of 1547 nm [219]. Different from previous work that used high-quality nanosheet-based TIs as mode-lockers, they used bulk-structured Bi${}_{2}$Te${}_{3}$ fabricated by ME and finally obtained 600-fs pulses with an average output power of 0.8 mW. The results illustrated that the bulk-structured TI could also be an efficient mode-locker with the advantages of low cost and easy fabrication. After that, Liu et al. used a Bi${}_{2}$Se${}_{3}$-PVA composite as SA in an anomalous dispersion fiber ring laser and achieved femtosecond mode-locked pulses of ∼660 fs with an SNR more than 55 dB, indicating its high stability [85].

Sotor et al. reported on the first stretched-pulse mode-locked laser based on TIs. In this work, they deposited bulk Sb${}_{2}$Te${}_{3}$ on the side-polished fiber and spliced it to a dispersion-managed laser resonator, which generated 128-fs pulses with an average output power of 1 mW [220]. In 2015, Yan et al. proposed a passive harmonic mode-locked fiber laser operating at 1562.4 nm enabled by a Bi${}_{2}$Te${}_{3}$ film SA, where the microfiber-based TI SA was fabricated by the PLD method for the first time [221]. As a result, stable fundamental mode-locking was demonstrated with a pulse width of 320 fs and an output power of 45.3 mW at the repetition rate of 2.95 GHz.

In 2015, the dissipative solitons operation of a fiber laser based on Sb${}_{2}$Te${}_{3}$ was proposed by Boguslawski et al. for the first time, which possessed a central wavelength of 1558 nm with a pulse duration of 167 fs and pulse energy of 0.21 nJ [222]. In 2019, Wei et al. reported a passively mode-locked all-fiber EDFL based on CVD-grown Bi${}_{2}$Te${}_{3}$ film with the maximum output power, as well as pulse energy of 40.37 mW and 23.9 nJ [223]. Later, Guo et al. employed CVD-grown Bi${}_{2}$Se${}_{3}$ as SA in a bidirectional pumped laser cavity, raising the parameters to 82.6 mW and 48.3 nJ [224] and 185.3 mW and 171.3 nJ [225], successively. The improvement of the parameters validated that CVD-grown Bi${}_{2}$Se${}_{3}$ exhibits a remarkable capability in high power mode-locked lasers.

In 2013, Luo et al. proposed the first passively Q-switched YDFL based on Bi${}_{2}$Se${}_{3}$ at 1067 nm, indicating that TIs SAs had come on the stage in ultrafast lasers at 1 μm [226]. In 2014, Li et al. achieved both Q-switched and Q-switched mode-locked operation in a solid-state laser in 1-μm band by using a Bi${}_{2}$Te${}_{3}$ SA mirror, obtaining the maximum output power of 183 and 247 mW, respectively [183]. Shortly afterward, Chi et al. reported on the generation of all-normal-dispersion dissipative-soliton pulses with a bulk Bi${}_{2}$Te${}_{3}$ at 1.06 μm [227]. At a repetition rate of 1.44 MHz, stable mode-locked pulses had a pulse duration of 230 ps. Equally important is that Dou et al. also exploited Bi${}_{2}$Se${}_{3}$ as SA in an all-normal-dispersion YDFL, which possessed a pulse width of 46 ps and maximum average output power of 33.7 mW at 44.6 MHz [88].

In 2015, Xu et al. achieved Q-switched operation at 1313 nm by using LPE-prepared Bi${}_{2}$Se${}_{3}$ nanosheets as SA and Nd:LiYF${}_{4}$ (YLF) crystals as the gain medium, obtaining pulse energy up to 1.23 μJ at a repetition rate of 161.3 kHz [228]. Later, Xu et al. proposed passively Q-switched mode-locked Nd:yttrium vanadate lasers enabled by large-size Bi${}_{2}$Te${}_{3}$ sheets at 1064 and 1342 nm separately both with a short pulse duration of a nanosecond, revealing the promising application of large-size Bi${}_{2}$Te${}_{3}$ in high-energy short pulse generation [229].

In 2018, Wang et al. demonstrated an all-solid-state mode-locked laser based on a large-area Bi${}_{2}$Te${}_{3}$ SA mirror prepared by the spinning coating-co-reduction approach (SCCA), which had a repetition rate up to 1 GHz, output power up to 180 mW, and SNR of 61 dB [230]. These results indicates that TIs could be potential SAs in solid-state lasers for the generation of highly stable ultrafast pulses.

In 2014, Luo et al. employed TIs as SA in a Q-switched TDFL at 1.98 μm for the first time, where TI-Bi${}_{2}$Se${}_{3}$ nanosheets were prepared by the LPE method [231]. Later, the first mode-locked fiber laser based on Bi${}_{2}$Te${}_{3}$ in the 2-μm region was obtained by Jung et al. [232]. They utilized the bulk-structured Bi${}_{2}$Te${}_{3}$ prepared by ME and achieved 795-fs pulses at a repetition rate of 27.9 MHz. In 2015, Yin et al. demonstrated the generation of stable bunched solitons and harmonically mode-locked solitons in a Bi${}_{2}$Te${}_{3}$-based mode-locked THDFL, obtaining a pulse duration of 1.26 ps at a repetition rate of 21.5 MHz [233].

Compared with Bi${}_{2}$Te${}_{3}$, the other commonly used TI (Bi${}_{2}$Se${}_{3}$) was applied to mode-locked fiber lasers in 2 μm very late until 2018, when Lee et al. proposed a mode-locked THDFL enabled by a bulk-structured Bi${}_{2}$Se${}_{3}$ with a pulse duration of ∼853 fs [234]. In the same year, Loiko et al. demonstrated a Q-switched thulium-doped gadolinium vanadate laser based on a Sb${}_{2}$Te${}_{3}$ film that was fabricated by the MSD method, obtaining a maximum average output power of 0.70 W at the repetition rate of ∼200 kHz [235].

In 2015, Yin et al. reported on the first mid-IR fiber laser mode-locked by Bi${}_{2}$Te${}_{3}$ SA mirror at 2830 nm with a pulse duration of ∼6 ps and maximum pulse energy up to 8.6 nJ [236]. Li et al. achieved passively Q-switched operation in a Ho-doped ZrF${}_{4}$-BaF${}_{2}$-LaF${}_{3}$-AlF${}_{3}$-NaF (ZBLAN) fiber laser induced by Bi${}_{2}$Te${}_{3}$ SA, which produced high-energy pulses with a maximum output power of 327.4 mW and pulse energy of 3.99 μJ [237]. This work indicated that TIs could be reliable SAs for mid-IR pulse generation.

In 2016, Tang et al. demonstrated a Q-switched Er-doped ZBLAN fiber laser by utilizing Bi${}_{2}$Te${}_{3}$ as SA, which possessed an average power up to 856 mW at the repetition rate of 92 kHz [238]. In 2018, Li et al. reported on a miniaturized all-fiber Ho-doped fiber laser (HDFL) Q-switched by Bi${}_{2}$Se${}_{3}$ nanosheets, in which the shortest pulse duration was 1.54 μs at the maximum output power of 315 μW [239].

As another 2D material widely applied in ultrafast photonics right after graphene, TIs enjoy various merits, including broadband saturable absorption properties, a large nonlinear refractive index, and an innate giant modulation depth (up to 95%) [217,240,241]. Therefore, TIs have been considered as remarkable SAs for the generation of ultrashort, high energy, and high repetition rate pulses within a wide wavelength ranging from visible to mid-IR regions [223,242,243,244]. However, the relatively slow relaxation time, complicated fabrication process, and relatively low mode-locking stability limit the applications of TIs in ultrafast photonics [49,70,245].

In addition, although Sb${}_{2}$Te${}_{3}$ SA demonstrated reliable performance in ultrashort and high power pulses generation [246,247], the majority of applications focus on the telecommunication band. Hence, it is worthwhile to explore more operating wavelengths of Sb${}_{2}$Te${}_{3}$ SA. Apart from that, more fabrication methods for TIs with lower cost and easier manipulation, as well as applications in a higher wavelength band of mid-IR region also need to be further studied.

### 3.3. Transition Metal Dichalcogenides

Since Wang et al. investigated the excellent saturable absorption properties of MoS${}_{2}$ at 800 nm [248], TMDs, such as MoS${}_{2}$, MoSe${}_{2}$, WS${}_{2}$, and WSe${}_{2}$, have come to the stage of ultrafast photonics applications. In 2014, Zhang et al. first reported on a mode-locked fiber laser at 1054.3 nm with a pulse width of 800 ps, in which a few-layer MoS${}_{2}$ film was fabricated through the hydrothermal exfoliation method [47]. Later, the first Q-switched fiber laser based on a MoS${}_{2}$-polymer composite SA was demonstrated by Woodward et al. obtaining a typical Q-switched pulse operated at 1068.2 nm with a pulse width of 2.7 μs [48]. Xu et al. realized a Q-switched solid-state laser by employing LPE-prepared MoS${}_{2}$ nanosheets in an Nd:yttrium aluminum perovskite (Nd:YAP) laser cavity, where high energy pulses with a peak power of 4.92 W were generated, revealing the promising potential of MoS${}_{2}$ SA for ultrafast solid-state lasers [249].

**Figure 10:**
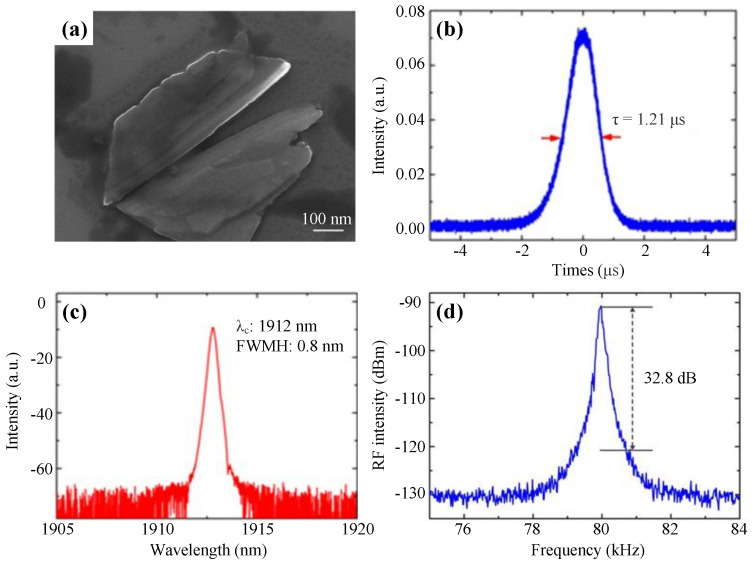
The experimental results of Q-switched YDFL enabled by WS${}_{2}$ SA. (**a**) SEM image of WS${}_{2}$ nanoplates. (**b**) Output spectrum, (**c**) single pulse profile, and (**d**) radio frequency (RF) spectrum at the repetition rate of 81.5 kHz. (**a**–**d**) adapted from [252].

In 2015, Zhao et al. proposed a simple chemical weathering-assisted exfoliation method to fabricate MoS${}_{2}$ and WS${}_{2}$ monolayers, which exhibited extraordinary performance in Q-switched and mode-locked all-solid-state lasers [250]. Based on this fabrication method, Hou et al. further disposed WS${}_{2}$ through ultrasonic treatment and used it as SA to obtain a mode-locked Yb:YAG laser with a pulse duration of 736 fs at a repetition rate of 86.7 MHz, further proving that WS${}_{2}$ could be an excellent SA for ultrafast solid-state lasers [251]. In the same year, Lin et al. demonstrated the first tunable Q-switched YDFL based on WS${}_{2}$ that prepared by the intercalation method, and the SEM image is shown in Figure 10a [252]. As shown in Figure 10b–d, the typical Q-switched pulses operated at 1048.1 nm with a pulse duration of 1.65 μs and an SNR of ∼50 dB at the repetition rate of 81.5 kHz.

In 2016, Cheng et al. reported on waveguide Nd:YAG lasers passively Q-switched by CVD-grown MoSe${}_{2}$ and WSe${}_{2}$ respectively, both achieving nanosecond pulse durations [253]. Adopting almost the same scheme, Wang et al. realized Q-switched Nd:YAG lasers based on WS${}_{2}$ solution of different concentrations [254]. By changing the concentrations of the WS${}_{2}$ solutions, they uncovered that the pulse width decreased and the repetition rate rose along with the increase of concentration.

In 2014, Liu et al. demonstrated a femtosecond mode-locked fiber laser at 1569.5 nm with a pulse duration of 710 fs, and a repetition rate of 12.09 MHz by exploiting a firmly MoS${}_{2}$-PVA composite as SA, which was the first application of MoS${}_{2}$ SA in a telecommunication band [255]. Following this work, Liu et al. realized a high-order passively harmonic EDFL mode-locked by microfiber-based MoS${}_{2}$ SA [256]. According to the results, the laser achieved highest harmonic order of 369th at the repetition rate of 2.5 GHz. Soon after that, stable Q-switched operation based on MoS${}_{2}$ with a widely tunable waveband from 1519.6 to 1567.7 nm was achieved by Huang et al. which possessed a maximum pulse energy of 160 nJ and an SNR of 50 dB [257].

Considering the defect of the polymer binder for decreasing the damage threshold, Khazaeizhad et al. coated the MoS${}_{2}$ film on a side-polished fiber to fabricate a polymer-free MoS${}_{2}$ SA. They used it to obtain not only dissipative soliton pulse trains with a bandwidth of 23.2 nm in the normal dispersion regime but also soliton-like pulses of 637 fs in the anomalous dispersion regime [258]. In addition to MoS${}_{2}$, WS${}_{2}$ is also used as a SA for generating ultrashort pulses. In 2015, Mao et al. uncovered the ultrafast saturable absorption property of WS${}_{2}$ nanosheets with the merit of a high damage threshold and used it as SA to achieve stable soliton mode-locking [259]. In pace with this work, stable Q-switched and femtosecond mode-locked operation in the EDFL based on LPE-grown WS${}_{2}$ were also reported by Kassani et al. and Khazaeinezhad et al. separately [260,261].

Yan et al. realized a harmonic mode-locked EDFL by inserting WS${}_{2}$ film-SA synthesized through the PLD method, where the 53rd harmonic with a pulse width of 452 fs and an SNR of 65 dB was obtained at the repetition rate of 1.04 GHz [262]. This work revealed that WS${}_{2}$ could be an outstanding mode-locker and Q-switcher in ultrafast photonics. In the same year, MoSe${}_{2}$ was first exploited as SA in 1.5 μm by Luo et al. who embedded few-layer MoSe${}_{2}$ in a polymer composite and achieved mode-locked soliton pulses with a pulse duration of 1.45 ps [263]. In 2016, Koo et al. utilized a MoSe${}_{2}$-PVA composite to demonstrate a femtosecond harmonic mode-locked fiber laser with a maximum harmonic order of 212th at the repetition rate of 3.27 GHz, which is the largest repetition rate achieved by 2D material-based lasers to date [264].

In 2017, Liu et al. transferred PLD-prepared WS${}_{2}$ on a small waist diameter tapered fiber along with a long fused zone covered by a gold film to obtain a SA that had a large modulation depth [265]. Thus, highly stable pulses with an SNR up to 93 dB and a pulse duration of 67 fs were obtained, which was the shortest pulse duration achieved in mode-locked fiber lasers based on TMDs, confirming the promising future of WS${}_{2}$ in ultrashort pulse generation. In 2018, a Q-switched EDFL based on CVD-grown MoSe${}_{2}$ was reported by Liu et al. which emitted 207-fs pulses with an SNR of 85 dB, further indicating that the combination of the CVD method and the tapered fiber structure is conducive to fabricate high performance SAs for the generation of stable ultrashort pulses [266].

In 2014, by introducing suitable defects in the process, Wang et al. fabricated a type of few-layered MoS${}_{2}$ with large broadband saturable absorption extending to 2.4 μm [267]. The MoS${}_{2}$ was applied to demonstrate Q-switching in solid-state lasers at 1.06, 1.42, and 2.1 μm separately. Later, Luo et al. reported on broadband Q-switched fiber lasers by using a few-layer MoS${}_{2}$-PVA composite SA at different wavelengths, where the span of the wavelengths covered from 1 to 2 μm [268]. Their work indicated unambiguously that few-layer MoS${}_{2}$ was a promising broadband SA. In 2015, Kong et al. realized both Q-switching and Q-switched mode-locking in a Tm:CLNGG laser induced by MoS${}_{2}$ golden mirror SA [269].

Subsequently, the first mode-locked operation based on MoS${}_{2}$ in 2 μm was achieved by Tian et al., who obtained a pulse energy of 15.5 nJ and a repetition rate of 9.67 MHz [270]. In the same year, Jung et al. proposed the first application of WS${}_{2}$ in an ultrafast mode-locked fiber laser at 1941 nm with an SNR up to 72 dB [271]. In 2017, Lee et al. achieved a femtosecond mode-locked fiber laser at 1912 nm based on MoSe${}_{2}$-PVA composite SA with a pulse duration of ∼920 fs and a repetition rate of 18.21 MHz [272]. Another relatively typical TMD, WSe${}_{2}$, was not used as a SA in the 2-μm region until 2018 by Wang et al., who demonstrated mode-locked soliton pulses had a pulse duration of 1.16 ps and an average output power of 32.5 mW at a repetition rate of 11.36 MHz [273].

In 2016, TMD SA started to be applied in the 3-μm region. Fan et al. reported on a Q-switched Er:lutetium oxide (Er:Lu${}_{2}$O${}_{3}$) laser at the central wavelength of 2.84 μm induced by a few-layer MoS${}_{2}$-based SA, which possessed a repetition rate of 121 kHz and pulse energy of 8.5 μJ, corresponding to a Watt-level average output power of 1.03 W [274]. Subsequently, Wei et al. achieved Q-switched operation in a fiber laser based on WS${}_{2}$ at 2865.7 nm [275]. The above are the early demonstrations of TMD SA for Q-switching in 3 μm, which indicated the potential of TMD SA applied in the mid-IR region.

In the next two years, Q-switched solid-state lasers in 3 μm based on MoS${}_{2}$ and WSe${}_{2}$ were demonstrated by Zhang and Liu et al. successively [276,277]. In 2020, Guo et al. achieved mode-locked operation based on CVD-grown WSe${}_{2}$ SA mirror at 2789.6 nm with a maximum average output power of 360 mW and a repetition rate of 42.43 MHz, which is the first time TMD severed as mode-locker in the 3-μm region [278].

Many merits of TMDs, such as layer-dependent bandgap and broadband operation, enable them as outstanding SAs from the visible to mid-IR regions [262,267], especially in the Q-switched operation [70,279]. However, the relatively large direct bandgap and low optical damage threshold limit the applications in long operating wavelength as well as in high power lasers [280]. Additionally, the commonly used methods for integrating TMD SAs thus far are either complex and difficult, or induce additional insertion loss to the cavity, which is worthy of optimization to achieve more fruitful results in the mid-IR region.

For example, Liu et al. prepared a WS${}_{2}$/SiO${}_{2}$ SA by the sol-gel method to overcome the shortcomings of scattering loss and a low optical damage threshold, as well as to achieve long-term stable mode-locked pulses under high power operation [281]. Furthermore, although MoS${}_{2}$, MoSe${}_{2}$, WS${}_{2}$, and WSe${}_{2}$ have been widely studied and applied, other TMDs, such as SnS${}_{2}$, SnSe${}_{2}$, and platinum diselenide, which show exceptional excellent properties, also deserve more investigation regarding ultrafast photonics [282,283,284,285,286].

### 3.4. Black Phosphorus

After the broadband nonlinear optical response of BP was reported in 2015 [82], Chen et al. utilized BP as SA to realize not only mode-locking at 1571.45 nm with a pulse duration down to 946 fs but also Q-switching at 1562.87 nm with a maximum pulse energy of 94.3 nJ [49]. Later, Li et al. achieved mode-locked operation based on BP with a pulse duration of 786 fs and Q-switched operation with a maximum pulse energy of 18 nJ in the telecommunication band [50]. They also studied the linear and nonlinear absorption properties of BP and found that it was polarization and thickness dependent.

In 2016, Chen et al. demonstrated the generation of stable mode-locked soliton pulses with a tunable wavelength extending from 1549 to 1575 nm by incorporating LPE-prepared BP into an all anomalous dispersion Er-doped cavity [287]. In the same year, Song et al. achieved a vector soliton fiber laser mode-locked by BP for the first time, where the LPE-prepared BP nanoflakes were transferred onto the end facet of a fiber [288]. According to the results, stable 670-fs soliton pulses centered at ∼1550 nm were obtained with a fundamental repetition rate of 8.77 MHz and an SNR of ∼60 dB.

In 2017, a dual-wavelength mode-locked vector soliton fiber laser based on few-layer BP centered at 1533 and 1558 nm with a pulse duration of ∼700 fs was proposed by Yun et al. which validated the potential of BP SA for ultrafast vector soliton generation [289]. In 2018, Jin et al. obtained stable stretched pulses in a mode-locked EDFL based on inkjet-printed BP with a shorter pulse duration of 102 fs and a wider bandwidth of 40 nm compared with previous reports. According to the results, the SNR was up to 60 dB, and the stable mode-locked operation could be maintained for a long time (>10 days), which indicated that the laser experiences long-term stability [290].

In 2015, Zhang et al. reported on a mode-locked solid-state laser based on BP SA mirror at 1064.1 nm with a pulse width of 6.1 ps and average output power of 460 mW [291]. In the same year, Ma et al. realized the first Q-switched solid-state laser enabled by BP at the central wavelength of 1046 nm where passively Q-switched pulses had an average output power of 37 mW at the maximum repetition rate of 113.6 kHz, corresponding to a pulse energy of ∼325.7 nJ [292]. Their works opened the way for BP SA in applications of ultrafast lasers in 1 μm.

In 2016, Al-Masoodi et al. demonstrated Q-switched operation in a BP-based YDFL, achieving a pulse energy of 328 nJ at the repetition rate of 32.9 kHz [293]. Then, Su et al. obtained femtosecond pulses of 272 fs in a solid-state laser mode-locked by BP for the first time with the maximum average output power as high as 0.82 W [294]. Rashidet et al. proposed a dual-wavelength Q-switched laser with a BP thin film operated at 1038.68 and 1042.05 nm [295]. In addition, Ahmad et al. proposed a wavelength-tunable BP-based Q-switched fiber laser at an operational wavelength ranging from 1056.6 to 1083.3 nm [296].

In 2017, Huang et al. achieved a Q-switched laser at 1064 nm where microfibers were sandwiched by BP flakes to integrate SA, providing an effective way to prolong the life of BP flakes by isolating them from the air [297]. In 2019, Wang et al. demonstrated both Q-switched and mode-locked lasers based on BP by inserting polarization-maintaining fiber Bragg gratings [298]. The operating wavelength could be turned to 1063.8 and 1064.1 nm, respectively, as well as concurrently.

**Figure 11:**
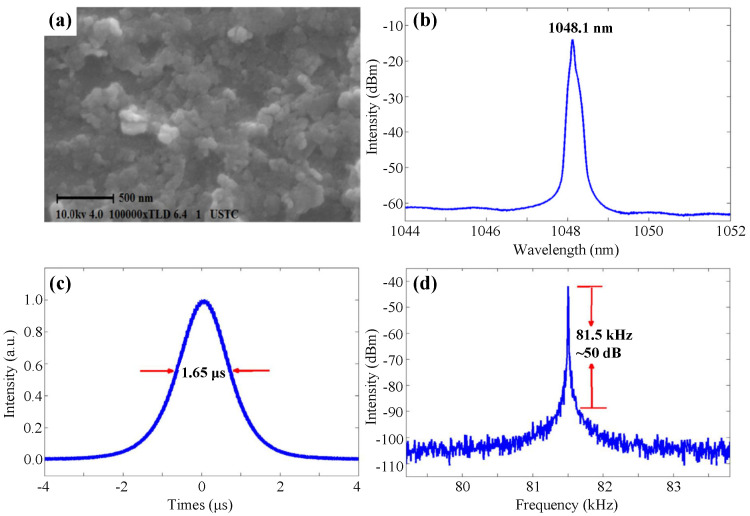
The experimental results of Q-switched THDFL enabled by BP SA. (**a**) SEM image of LPE-grown BP nanoplatelets. (**b**) Single pulse profile, (**c**) output spectrum, and (**d**) RF spectrum at a repetition rate of 113.3 kHz. (**a**–**d**) adapted from [299].

Following the initial applications in 1 μm, BP began to be used in the eye-safe region of 2 μm. In 2015, Sotor et al. first reported on a mode-locked fiber laser enabled by mechanically exfoliated BP films at 1910 nm with a pulse duration of 739 ps and an average output power of 1.5 mW at a repetition rate of 36.8 MHz [181]. In 2016, Yu et al. achieved the first Q-switched operation in 2 μm based on BP with the shortest pulse width of 731 ns. Figure 11a shows the SEM image of the LPE-prepared BP. As shown in Figure 11b–d, the laser produced 1.21-μs pulses with the central wavelength of 1912 nm and an SNR of 32.8 dB at the repetition rate of 79.8 kHz [299].

Later, the earliest solid-state laser Q-switched by BP in 2 μm was demonstrated by Chu et al., who achieved the shortest pulse duration of 1.78 μs at the repetition rate of 19.25 kHz, corresponding to a pulse energy of 7.84 μJ [300]. Then, Zhang et al. reported on a dual-wavelength Q-switched Tm:YAP bulk laser operating at 1969 and 1979 nm, which further proved the capability of BP for Q-switched operation in 2 μm [301]. When it came to 2017, Pawliszewska et al. proposed the first use of BP SA as a mode-locker in HDFL, where harmonic operation up to 10th order was demonstrated with a pulse duration of 1.3 ps at the repetition rate of 290 MHz [302].

In 2015, Qin et al. demonstrated Q-switched operation at 2.8 μm based on a BP SA mirror for the first time obtaining a maximum average output power of 485 mW and pulse energy of 7.7 μJ [81]. Without stopping, in the next year, they adopted the same method to fabricate BP SA and obtained a mode-locked Er-doped ZBLAN fiber laser with a pulse duration of 42 ps and a repetition rate of 24 MHz, which promoted the applications of BP as SA for ultrafast lasers in the mid-IR region [303]. In 2016, Kong et al. reported on a BP-based Q-switched Er:Y${}_{2}$O${}_{3}$ ceramic laser at 2.72 μm with a pulse energy of 0.48 μJ at 12.6 kHz [304].

Soon after that, Li et al. inserted LPE-prepared BP into rare earth ion doped fluoride fiber lasers to realize mode-locked and Q-switched operation, respectively, thus, further enhancing the working wavelength of BP-based fiber lasers [80]. In the same year, a dual-wavelength Q-switched solid-state laser at 2.79 μm was demonstrated by Liu et al. [305]. They adopted BP nanoflakes as SA in an Er:strontium fluoride (Er:SrF${}_{2}$) bulk laser that generated 702-ns pulses at a repetition rate of 77.03 kHz.

In 2018, Qin et al. demonstrated both mode-locked and Q-switched lasers in 3.5 μm enabled by BP SA, which was the first time mode-locking and Q-switching are operated in such a high spectral region, validating the superb potential of BP as SA in the mid-IR region. In 2019, Woodward et al. reported on a dysprosium-doped fiber laser Q-switched by BP SA with a tunable wavelength from 2.97 to 3.32 μm by using an acousto-optic tunable filter [306].

Benefiting from the layer-dependent direct bandgap that can be broadly turned from ∼0.3 (bulk) to ∼2 eV (monolayer), BP stands out as an excellent filler between zero-bandgap graphene and wide-bandgap TMDs [307,308,309]. Although BP can be fabricated conveniently with low cost [81], its instability in air and water molecules requires relatively strict conditions for its preparation and operation, which restricts the application especially in high power regimes [310,311,312]. For now, there is a need for more research on not only economic fabrication methods for stable BP SA but also its application in certain types of ultrafast lasers; for example, harmonic mode-locked lasers with high repetition rate pulses, which possess critical applications in optical communication [313], spectroscopy [43], and frequency bombing [314].

### 3.5. MXenes

Since Naguib et al. first exfoliated Ti${}_{3}$C${}_{2}$ nanosheets from Ti${}_{3}$AlC${}_{2}$, MXenes joined the 2D materials family [51,116]. In 2017, Jhon et al. utilized Ti${}_{3}$CNT${}_{x}$ monolayers to obtain both stable Q-switched and mode-locked pulses at 1558 and 1557 nm [315]. According to the experimental results, a EDFL generated a pulse width of 660 fs, and the main properties were well retained from the Ti${}_{3}$CNT${}_{x}$ monolayers to stacked ones, which indicated that femtosecond pulses can be demonstrated conveniently by using MXenes as SA without the troublesome process of monolayer dispersion.

In the same year, Jiang et al. discovered the broadband light signal manipulating capabilities of Ti${}_{3}$C${}_{2}$T${}_{x}$ films and deposited them onto a side-polished fiber to obtain a mode-locked EDFL with a pulse duration of 159 fs [316]. In 2018, Kim et al. realized a passively mode-locked laser at 1556 nm based on Ti${}_{3}$C${}_{2}$T${}_{x}$ fabricated by the AAE method, where a soliton pulse with a pulse width of 800 fs was generated at a repetition rate of 6.22 MHz [317]. In 2019, Li et al. reported on a mode-locked EDFL based on Ti${}_{3}$C${}_{2}$T${}_{x}$, which was also synthesized by the AAE method [318].

**Figure 12:**
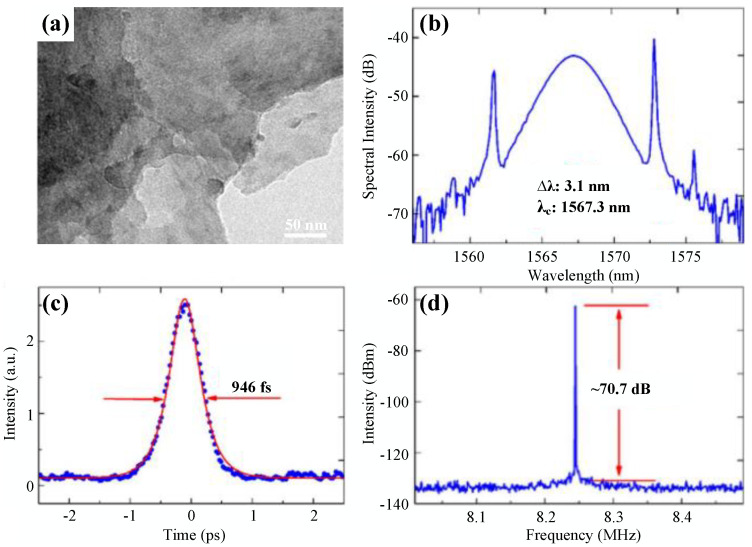
The experimental results of mode-locked EDFL enabled by MXene SA. (**a**) TEM image of the Ti${}_{3}$C${}_{2}$T${}_{x}$ flakes. (**b**) Output spectrum. (**c**) Autocorrelation trace. (**d**) RF spectrum. (**a**–**d**) adapted from [318].

Figure 12a shows the SEM image of the Ti${}_{3}$C${}_{2}$T${}_{x}$ flakes. As shown in Figure 12b–d, the laser yielded 946-fs pulses at 1567.3 nm with a bandwidth of 3.1 nm and an SNR up to 70.7 dB, which meant the pulse generation was highly stable. In the same year, Feng et al. demonstrated a 39th order harmonic mode-locked EDFL enabled by Ti${}_{3}$C${}_{2}$T${}_{x}$ SA for the first time [319]. The pulse width was 850 fs, and the repetition rate was up to 218.4 MHz with high commercial value in certain fields, such as electronic communication. Later, Yi et al. investigated the broadband SA properties of Ti${}_{2}$CT${}_{x}$ and incorporated the solution-processed Ti${}_{2}$CT${}_{x}$ onto a D-shape fiber to obtain a mode-locked pulse at 1565 nm with a pulse duration of 5.3 ps [56].

Wu et al. proposed a dispersion-managed fiber laser mode-locked by microfiber-based Ti${}_{3}$C${}_{2}$T${}_{x}$ SA with a stretched pulse of 104 fs [320]. In 2020, Huang et al. realized the highest harmonic MXene-based mode-locked fiber laser so far, where 206th harmonic order pulses at 1559.1 nm were generated with a pulse width of 940 fs and a high repetition rate of 1.01 GHz [321]. Li et al. first achieved a square-shaped mode-locked fiber laser based on Nb${}_{2}$C, which was fabricated through the MSD technique [322]. In 2021, Jafry et al. demonstrated mode-locked EDFLs with thin films of Ti${}_{3}$C${}_{2}$T${}_{x}$ and Ti${}_{3}$C${}_{2}$T${}_{x}$, separately, achieving the highest pulse energies (6.03 and 7.69 nJ) generated by MXene-based SA up to now [323].

In 2018, Jiang et al. obtained an ultrafast fiber laser mode-locked by few-layer Ti${}_{3}$C${}_{2}$T${}_{x}$ deposited onto a side-polished fiber at 1065.89 nm, which possessed a pulse duration of 480 ps, repetition rate of 18.96 MHz, and pulse energy of 0.47 nJ [316]. In the same year, Feng et al. reported on a Q-switched Nd:YAG ceramic laser enabled by Ti${}_{3}$C${}_{2}$T${}_{x}$ at 1064 nm, which was the first time a Q-switched solid-state laser in the 1-μm region with MXene SA had been achieved [324]. At a repetition rate of 186 kHz, the shortest pulse width was 359 ns and the maximum pulse energy was 0.66 μJ. Soon after that, the first mode-locked solid-state laser with MXene was realized by Sun et al. where LPE-prepared Ti${}_{3}$C${}_{2}$T${}_{x}$ was implemented into a Yb:potassium yttrium tungstate (Yb:KYW) laser, and 316-fs pulses at a repetition rate of 64.06 MHz were produced [325].

In 2020, Shi et al. obtained a stable mode-locked fiber laser based on Ti${}_{2}$CT${}_{x}$ nanosheets with an SNR up to 75 dB [326]. Subsequently, Ma et al. demonstrated a hybrid mode-locked fiber laser by employing nonlinear polarization evolution to YDFL together with vanadium carbide MXene (V${}_{2}$CT${}_{x}$) nanosheets, enhancing the output performance regarding the pulse stability and cavity efficiency [327]. Self-starting stable 72-fs pulses with a high pulse energy of 1 nJ were produced in the laser cavity, which is the shortest pulse duration from MXene-based lasers. The experimental results confirmed that V${}_{2}$CT${}_{x}$ SA is promising in ultrashort pulse generation with high energy.

In 2017, Jhon et al. reported on a passively Q-switched THDFL by using Ti${}_{3}$CNT${}_{x}$ as SA at the central wavelength of 1875 nm, indicating that the operating range of MXenes could be further extended [315]. In 2019, Zu et al. demonstrated a solid-state laser Q-switched by MXene SA in 2 μm, where the AAE-processed Ti${}_{3}$C${}_{2}$T${}_{x}$ was integrated with Tm, Gadolinium doped Calcium fluoride crystal and a pulse width of 2.39 μs at the repetition rate of 19.61 kHz was obtained [328]. In this year, the first self-starting mode-locked TDFL with Ti${}_{3}$C${}_{2}$T${}_{x}$ SA was reported by Jiang et al. at 1862 nm with a pulse duration of 2.11 ps and a repetition rate of 13.45 MHz, which further showed the applicability of MXene SA in broadband operation [329].

In 2020, Wang et al. demonstrated a mode-locked fiber laser based on Ti${}_{3}$C${}_{2}$, where the vector solitons were generated at the central wavelength of 1965 nm with a 19th harmonic pulse train [330]. They also obtained noise-like pulses evolving from vector solitons with a bandwidth of 3.3 nm by changing the polarization in the cavity, and made the first contribution to explorations of the polarization-independent property of Ti${}_{3}$C${}_{2}$. Later, Ahmad et al. realized a Ti${}_{3}$C${}_{2}$T${}_{x}$-based passively Q-switched fiber laser with a widely tunable wavelength ranging from 1895 to 2050 nm [331].

In 2021, Gao et al. proposed a passively mode-locked fiber laser by depositing Nb${}_{2}$C on the tapered fiber, achieving a maximum harmonic order of 69th at the repetition rate of 411 MHz [332]. The results suggested the potential of Nb${}_{2}$C SA in the mode-locked device. Soon after that, Niu et al. demonstrated a doubly Q-switched Tm:lutetium aluminum garnet (Tm:LuAG) laser by using Ti${}_{3}$C${}_{2}$T${}_{x}$ SA and an acousto-optic modulator, which possessed a shorter pulse width of 178 ns and higher pulse peak power of 1062 W compared with the singly Q-switched laser [333].

In 2019, Zhou et al. obtained passively Q-switched pulses in 3 μm based on Ti${}_{3}$C${}_{2}$T${}_{x}$ with a maximum output power of 517 mW at the repetition rate of 78.12 kHz, which carved the way for the MXene used in mid-IR pulse generation [334]. Later, Yi et al. realized a Q-switched Er-doped ZBLAN fiber laser based on Ti${}_{2}$CT${}_{x}$, obtaining a pulse duration of 730 ns and repetition rate of 99.5 kHz [56]. Subsequently, Bharathan et al. reported on a mid-IR fiber laser mode-locked by MXene SA operated near 2.8 μm, which had a pulse width of 223.7-ns and an average power of 223 mW [335].

In 2020, the first Watt-level 2D material-based Q-switched fiber laser in the mid-IR region was reported by Wei et al., who achieved a maximum average output power of 1.09 W by using Ti${}_{3}$C${}_{2}$T${}_{x}$ as the SA [336]. The study demonstrated that Ti${}_{3}$C${}_{2}$T${}_{x}$ is an outstanding SA for mid-IR pulse generation with high power. In 2021, Feng et al. proposed an Er:Lu${}_{2}$O${}_{3}$ laser Q-switched by LPE-prepared Nb${}_{2}$CT${}_{x}$ nanosheets [337]. According to the experimental results, the Q-switched laser could generate a laser pulse of 223.7 ns, which is the shortest pulse duration of MXene-based Q-switched lasers in 3-μm region to date.

As a kind of novel 2D material, MXenes have become a hotspot as prominent SAs in recent years accounting for the excellent damage threshold, ultrafast recovery time, and broadband operation [56,318,338]. Currently, MXene-SAs have achieved mode-locking and Q-switching in a broad waveband from the visible to mid-IR regions, excelling at the generation of ultrashort pulses with good stability and high energy. However, the operating wavelength is still limited to under 3 μm, and the applications of various MXene-SAs in each waveband are uneven, which mainly focus on Ti${}_{3}$C${}_{2}$T${}_{x}$ and Ti${}_{3}$C${}_{2}$. Therefore, more efforts should be taken to expand the operating wavelength of MXene-based lasers. Meanwhile, more exciting properties and applications in ultrafast lasers of other MXenes, such as Nb${}_{2}$CT${}_{x}$ and V${}_{2}$CT${}_{x}$, are worth exploring further.

### 3.6. Heterostructures

**Figure 13:**
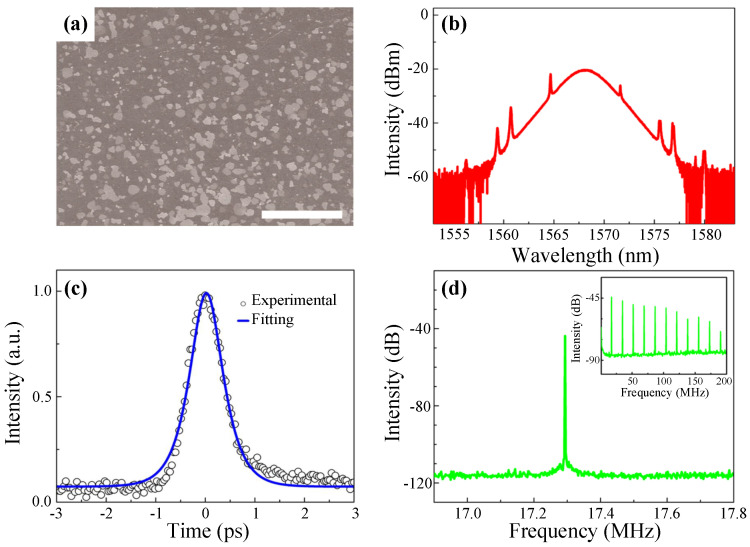
The experimental results of mode-locked fiber laser enabled by graphene/Bi${}_{2}$Te${}_{3}$ heterostructure SA. (**a**) SEM image of graphene/Bi${}_{2}$Te${}_{3}$ heterostructure with 15% coverage of Bi${}_{2}$Te${}_{3}$. (**b**) Output spectrum. (**c**) Autocorrelation trace. (**d**) RF spectrum (insert: RF spectrum with a wideband up to 200 MHz). (**a**–**d**) adapted from [340].

In order to improve the performance of SAs based on 2D materials, researchers combined the advantages of different 2D materials by forming van der Waals heterostructures, which exhibits superior potential in applications of ultrafast photonics [339]. In 2015, Mu et al. first demonstrated Q-switched and mode-locked fiber lasers based on graphene/Bi${}_{2}$Te${}_{3}$ heterostructure SA [340]. The SEM image of the heterostructure is shown in Figure 13a. Mode-locked pulses centered at 1568.07 nm with a pulse duration of 837 fs, repetition rate of 17.3 MHz, and an SNR of 60.7 dB are shown in Figure 13b–d. According to the results, they found that the heterostructure combined the advantages of high modulation depth of Bi${}_{2}$Te${}_{3}$ and fast carrier dynamics of graphene, which can be tunable by controlling the coverage of Bi${}_{2}$Te${}_{3}$ on graphene.

Later, Jiang et al. used MoS${}_{2}$/graphene nanocomposites as SA in EDFLs, where both mode-locked and Q-switched operations were achieved [341]. From then on, various heterostructure SAs based on different 2D materials started sparking in ultrafast photonics. In 2017, Liu et al. fabricated a graphene/BP heterostructure to overcome the instability of BP in the air [342]. Furthermore, they used it as a SA to realize not only high energy Q-switching of 267.5 nJ but also mode-locking with a 148-fs pulse width, which exhibited better performance than the laser based on single graphene or BP. Li et al. reported a Q-switched waveguide laser with a maximum output power of 275 mW in 1-μm band, in which the graphene/WS${}_{2}$ heterostructure was used to enhance the output pulse energy [343]. Tan et al. proposed an Nd:YAG ceramic waveguide laser based on a graphene/WSe${}_{2}$ heterostructure that was modified by ion beam technology [344].

You et al. proposed passively Q-switched solid-state lasers in 2- and 3-μm bands based on a Bi${}_{2}$Te${}_{3}$/graphene heterostructure [345]. The presence of graphene made up for the defect of easy oxidation and the low thermal conductivity of Bi${}_{2}$Te${}_{3}$, revealing the feasibility of Bi${}_{2}$Te${}_{3}$/graphene SA to generate high energy pulses at a long wavelength. Zhao et al. demonstrated Q-switched operation at the wavelength of 2.8 μm based on MoS${}_{2}$/graphene heterostructure that was prepared through the hydrothermal method, thus, further promoting the applications of heterostructure SA in the mid-IR region [346]. Chen et al. applied WS${}_{2}$-MoS${}_{2}$-WS${}_{2}$ heterostructure into mode-locked EDFLs and obtained 296-fs pulses with an SNR up to 90.3 dB, which revealed the capability of TMD-based heterostructure SA in ultrafast applications [147].

In 2018, Liu et al. achieved both mode-locking and Q-switching based on a MoS${}_{2}$-Sb${}_{2}$Te${}_{3}$-MoS${}_{2}$ heterostructure SA with a large modulation depth and high damage threshold [347]. Xue et al. fabricated a MoS${}_{2}$/BP heterostructure SA through LPE and the spin-coating method [348]. They adopted it in a Tm:YAP laser and realized Q-switched operation with an average output power of 3.6 W and pulse energy of 41.8 nJ, validating its excellent saturable absorption properties. In 2019, MoS${}_{2}$/WS${}_{2}$ heterostructure SA was asserted in an EDFL by Liu et al. where 154-fs pulses with a maximum output power of 19.8 mW and an SNR of 91.2 dB were generated at a repetition rate of 74.6 MHz [349].

In 2021, Xia et al. combined the advantages of BP and MXene to fabricate the BP/Ti${}_{3}$C${}_{2}$ heterojunction, which possessed a large modulation depth of 58.2% and a small saturation intensity of 1.13 GW/cm${}^{2}$ in the communication band [350]. They further proved the applicability of BP/Ti${}_{3}$C${}_{2}$ heterojunction in ultrafast photonics by inducing it as a SA in a mode-locked EDFL. As a result, a 753-fs pulse width was achieved at 11.7 MHz with a high SNR of 75 dB, and a harmonic mode-locked operation up to the 51st order was also realized at 588 MHz.

In general, 2D material-based heterostructure SAs have attracted wide attention in ultrafast photonics due to excellent merits of modifying the nonlinear optical properties and broadening the response wavelength range [190,340,347,350,351,352]. Moreover, heterostructures offer a prominent alternative for optoelectronic devices to achieve desirable performance, such as photodetectors, lithium-ion batteries, and field-effect transistors [352,353,354,355,356,357,358,359].

Currently, the operated wavelengths of lasers enabled by heterostructure SAs mainly focus on the 1- and 1.5-μm bands. Graphene and TMD are the most commonly used 2D materials to form heterostructure SAs in ultrafast lasers, while the novel MXenes are relatively rarely used. Therefore, in order to achieve higher performance ultrafast lasers, it is necessary to explore the heterostructure SAs based on MXenes and other 2D materials.

Ultrafast lasers have attracted wide attention owing to the generation of ultrashort pulses with high peak power and pulse energy. The emergency of 2D material SAs has brought bright prospects for ultrafast lasers, which are key components to realize high-performance mode-locked and Q-switched operation [43]. Table 1 summarizes the recent developments of ultrafast lasers based on typical 2D material SAs. Ultrafast pulsed lasers have been mostly investigated in the 1-, 1.5- and 2-μm regions, even up to the mid-IR region of 3 μm. Different gain media are selected in these lasers to obtain pulses centered at diverse wavelengths. Yb-doped fiber, Nd:YAG, and Yb:YAF crystals are commonly used as gain media in 1 μm, while Er-doped fiber, Er:YAG, and Cr:YAG crystals are widely utilized to achieve ultrafast pulses at the wavelength of 1.5 μm.

In the case of the 2-μm region, researchers tend to employ Tm-doped, Tm-Ho co-doped fibers, and Tm:YAG crystals to obtain ultrafast pulses. In order to realize ultrafast pulsed lasers in the mid-IR region, the ZBLAN fiber is the general gain medium in laser cavities. Ultrafast pulsed lasers based on 2D materials, such as graphene, TIs, TMDs, BP, MXenes, and their heterostructures, can be achieved in 1, 1.5, 2, and 3 μm with corresponding gain media. Moreover, the performances of ultrafast pulsed lasers using 2D materials as SAs are generally outstanding, including a short pulse width, high repetition rate, large pulse energy, and high stability. Each 2D material possesses unique properties, which results in superior output performances of ultrafast pulses at different wavelengths.

Short pulse width is one of the most remarkable merits of ultrafast lasers with 2D materials. In the telecommunication band, the pulse width could achieve the order of femtosecond in mode-locking. For instance, mode-locked pulses with the pulse widths of 870, 128, 163.5, 102, and 159 fs could be generated from EDFLs without amplification or compression based on graphene, TIs, TMDs, BP, and MXenes, respectively. A high repetition rate is another superior output performance, which can be realized by harmonic mode-locked operation. Sobon et al. used graphene to achieve harmonic mode-locked EDFL with the repetition rate of 2.22 GHz [196]. A 2.95-GHz repetition rate was obtained in a harmonic mode-locked fiber laser with TI-Bi${}_{2}$Te${}_{3}$ centered at 1.5 μm [221]. With a TMD-WS${}_{2}$ film SA in a fiber laser, Yan et al. obtained a repetition rate up to 1 GHz at the wavelength of 1559.7 nm [360].

In the next year, Koo et al. raised the repetition rate up to 3.27 GHz by integrating MoSe${}_{2}$-PVA SA into EDFL [264]. In 2020, Huang et al. realized a harmonic mode-locked fiber laser with Ti${}_{3}$C${}_{2}$T${}_{x}$ up to 1.01 GHz, which is the highest repetition rate of MXene-based lasers to date [321]. The above experimental results indicated that graphene and TIs are promising candidates for generating harmonic mode-locked pulses with a high repetition rate. In addition, ultrafast lasers based on BP have not achieved a repetition rate up to the order of GHz, which are worthy of further investigations. Compared with mode-locking, Q-switching more is easier to realize ultrafast pulsed lasers with high pulse energy and output power. Li et al. utilized Bi${}_{2}$Te${}_{3}$ to obtain a Q-switched fiber laser in the 3-μm region, generating 3.99 μJ-pulses with the output power of 327.4 mW [237]. Wei et al. reported on a MXene-based Er-doped ZBLAN fiber laser with the maximum pulse energy of 28 μJ and the output power of 1.9 W [336]. However, the output power and pulse energy of graphene and TMDs in Q-switched fiber lasers are commonly below 150 mW and 2 μJ [361,362]. Moreover, a BP-based Q-switched laser at 3 μm was achieved with a pulse energy of 7.7 μJ and output power of 485 mW, whereas its easy oxidation under high laser excitation limits the applications [81,288,363]. Hence, TIs and MXenes are expected to achieve pulses with high pulse energy and output power in mid-IR region.

2D materials possess various characteristics and diverse preparation methods, which have an impact on the output performance of ultrafast pulsed lasers. Lasers enabled by graphene cover a wide waveband from the visible to mid-IR regions, which is fabricated mostly by ME and CVD methods [196,201,208,214,364]. Although TIs can be applied for broadband operation, the operating wavelengths of mode-locked lasers based on TIs focus on the telecommunication band, indicating that more applications in other wavebands need to be studied [88,220,221,234]. CVD and LPE are typical methods to fabricate TMDs that always serve as Q-switchers in near-infrared lasers.

However, the investigations of mode-locked lasers based on TMDs in the mid-IR region are restricted by the large direct bandgap in the monolayer state and low damage threshold [280,365,366]. BP not only enjoys broadband operation up to 3.5 μm but is capable of achieving femtosecond mode-locked pulses from the 1- to 2-μm regions as well [181,191,294,367]. Although BP can be easily prepared by ME or LPE methods, the easy oxidation in the air requires strict conditions for its preparation and operation [310,311,312].

**Table 1:** Summary of ultrafast lasers based on 2D material SAs. Note that the results obtained from different conditions including various 2D materials, gain media, and experimental purposes.

2D Materials		Fabrication Methods	Gain Media	λ (nm)	τ (s)	$f_{\mathrm{rep}}$ (Hz)	Energy (nJ)	Power (mW)	SNR (dB)	Ref.
Graphene	Graphene	CVD	Alexandrite	750	65 f	5.56 M	1.4	8	75	[364]
	Graphene	CVD	Yb	1069.8	580 p	0.9 M	0.41	0.37	70	[201]
	Graphene	MBE	Nd:YAG	1064	161 n	660 k	159.2	105	–	[200]
	Graphene	LPE	Er	1525	∼1.15 p	∼8 M	∼125 p	∼1	∼80	[188]
	Graphene	Modified Hummers	Er	1566.2, 1566.4	3.7 μ	3.3–65.9 k	16.7	1.1	–	[39]
	Graphene	ME	Er	1560.5	900 f	2.22 G	4.3 p	9.6	50	[196]
	Graphene	LPE	Er	1550	∼29 f	18.67 M	2.8	52	62	[368]
	Graphene	CVD	Tm:CLNGG	2018	729 f	98.7 M	0.6	60.2	–	[206]
	Graphene	CVD	Tm	2007.1, 2010.4	1.4 μ	44–53 k	85	4.5	40	[208]
	Graphene	LPE	Tm	1940	3.6 p	6.46 M	∼0.4	∼2	∼70	[207]
	Graphene	CVD	Tm	1897.7	122 n	0.964 M	35.2	34	67	[209]
	Graphene	CVD	Yb	1035	6.5 n	16.29 M	0.81	13	55	[55]
			Er	1564	870 f	19.30 M	10.4 p	0.2	64	
			Tm-Ho	1908	65 n	1.82 M	16.2	29	60	
	Graphene	–	Er-doped ZBLAN	2783	2.9 μ	18.9–37.2 k	0.74–1.67 μ	15–62	30	[361]
	Graphene	CVD	Er-doped ZBLAN	2784.5	42 p	25.4 M	0.7	18	43.5	[214]
TIs	Bi${}_{2}$Se${}_{3}$	LPE	Pr-doped ZBLAN	635.5, 635.7	244–761 n	191.6–454.5 k	22.3	7.6	∼43	[243]
	Bi${}_{2}$Se${}_{3}$	LPE	Yb	1067.66	1.95 μ	8.3–29.1 k	17.9	0.46	48	[226]
	Bi${}_{2}$Se${}_{3}$	PM	Yb	1031.7	46 p	44.6 M	0.756	33.7	58	[88]
	Bi${}_{2}$Se${}_{3}$	PM	Er	1565.1	14 μ	8.865 k	12.6	112 μ	–	[86]
	Bi${}_{2}$Se${}_{3}$	ME	Tm-Ho	1912.12	853 f	∼18.37 M	–	–	65	[234]
	Bi${}_{2}$Se${}_{3}$	LPE	Tm	1980	4.18 μ	8.4–26.8 k	313	8.4	43	[231]
	Bi${}_{2}$Se${}_{3}$	LPE	Ho-doped ZBLAN	2920.6	1.54–3.47 μ	24.97–56.13 k	6	315 μ	∼43	[239]
	Bi${}_{2}$Te${}_{3}$	ME	Yb	1057.82	230 p	1.44 M	0.599	0.86	∼77	[227]
	Bi${}_{2}$Te${}_{3}$	Spin coating- co-reduction	Nd:YVO${}_{2}$	1064	7.9 p	949 M	0.1 n	181	61	[230]
	Bi${}_{2}$Te${}_{3}$	ME	Nd:YVO${}_{3}$	1064	97 n	47 k	0.6 μ	26.1	–	[229]
			Nd:YVO${}_{4}$	1342	93 n	75 k	0.4 μ	33.2		
	Bi${}_{2}$Te${}_{3}$	HI/E	Er:YAG	1645	6.3 μ	40.7 k	5.3 μ	210	–	[46]
	Bi${}_{2}$Te${}_{3}$	LPE	Er	1566.9	14 μ	6.97 k	–	–	36.4	[369]
	Bi${}_{2}$Te${}_{3}$	PLD	Er	1562.4	920 f	2.95 G	15.4 p	45.3	60	[221]
	Bi${}_{2}$Te${}_{3}$	HI/E	Ho-doped ZBLAN	2979.9	1.37–4.83 μ	46.20–81.96 k	3.99 μ	327.38	37.4	[237]
	Sb${}_{2}$Te${}_{3}$	ME	Er	1565	128 f	22.32 M	44.8 p	1	65	[220]
	Sb${}_{2}$Te${}_{3}$	MSD	Er	1558	167 f	25.38 M	0.21	5.34	68	[222]
	CoSb${}_{3}$	–	Er	1557.9	∼833 f	14.48 M	6.9 p	0.1	57	[370]
TMDs	MoS${}_{2}$	HI/E	Yb	1054.3	800 p	6.58 M	1.41	9.3	50	[47]
	MoS${}_{2}$	LPE	Nd:YAP	1079.5	227–580 n	32–232.5 k	1.11 μ	0.26	–	[254]
	MoS${}_{2}$	CVD	Er	1568	4.98 p	26.02 M	–	–	63	[258]
			Er	1568	637 f	33.48 M			61		
	MoS${}_{2}$	LPE	Er	1551.2	5.7 μ	16.78 k	–	–	∼50	[257]
	MoS${}_{2}$	LPE	Yb	1066.5	5.8 μ	6.4–28.9 k	32.6	0.9	44.6	[268]
			Er	1565	5.4–23.2 μ	6.5–27.0 k	63.2	1.7	54.5		
			Tm	2032	1.76 μ	33.6–48.1 k	∼1 μ	47.3	54.6		
	MoS${}_{2}$	LPE	Er-doped ZBLAN	2754	1.84 μ–806 n	36–70 k	1–2 μ	37–140	40	[362]
	WS${}_{2}$	Chemical weathering exfoliation	Yb:YAG	1057.5	736 f	86.7 M	3.11	27	∼51	[251]
	WS${}_{2}$	LPE	Nd:YAG	1064.5	1.28–2.36 μ	28.59–45.25 k	1.2 μ	54	–	[254]
	WS${}_{2}$	CVD	Er	1557.4	163.5 f	63.133 M	0.45	28.5	96	[371]
	WS${}_{2}$	PLD	Er	1559.7	452 f	1.04 G	10.87 p	11.3	48	[360]
	WS${}_{2}$	LPE	Tm-Ho	1941	1.3 p	34.8 M	∼17.2 p	∼0.6	72	[271]
	WS${}_{2}$	Sulfidation grown	Ho/Pr co-doped ZBLAN	2865.7	1.73–3.8 μ	25.6–131.6 k	0.17–0.42 μ	4–48.4	40.5	[275]
BP	BP	LPE	Yb:CYA	1064	620 n–1.2 μ	∼87.7–113.6 k	182.4–325.7	16-37	–	[292]
	MoSe${}_{2}$	CVD	Nd:YAG	1064	80–290 n	0.995–3.334 M	35.9	115.1	–	[253]
	WSe${}_{2}$	CVD	Nd:YAG	1064	52–400 n	0.781–2.938 M	19	45.7	–	
	WSe${}_{2}$	CVD	Tm	1863.96	1.16 p	11.36 M	2.89	32.5	53	[273]
	PtSe${}_{2}$	CVD	Ho/Pr co-doped ZBLAN	2865	620 n–1.72 μ	104.2–238.1 k	0.14–0.39 μ	15-93	30	[372]
	BP	LPE	Yb, Lu:CALGO	1053.4	272 f	63.3 M	6.48	0.82	∼62	[294]
	BP	ME	Yb	1038.7, 1042.1	1.16–2.05 μ	52.52–58.73 k	2.09	0.12	50	[295]
	BP	ME	Er	1532.5	9.5–3.1 μ	26–40 k	∼18.6	728 μ	–	[50]
	BP	ME	Er	1558.7	∼786 f	14.7 M	∼0.1	1.6	∼56	
	BP	LPE	Er	1555	102 f	23.9 M	71 p	1.7	60	[290]
	BP	ME	Tm	1910	739 f	36.8 M	40.7 p	1.5	70	[181]
	BP	LPE	Tm-Ho	1912	731 n–1.42 μ	69.4–113.3 k	632.4	71.7	32.8	[299]
	BP	LPE	Tm:YAP	1969, 1979	181–720 n	∼41–81 k	39.5 μ	3100	–	[301]
	BP	LPE	Tm:CYA	1029	1.73 μ	63.9 k	0.09 μ	6	–	[304]
			Tm:CYA	1930	3.7 μ	17.7 k	0.68 μ	12		
			Er:Y${}_{2}$O${}_{3}$	2720	4.47 μ	12.6 k	0.48 μ	6		
	BP	ME	Er-doped ZBLAN	2783	42 p	24 M	25.5	613	60	[303]
	BP	LPE	Er-doped ZBLAN	3462	2.05 μ	66.3 k	1.8 μ	120	–	[367]
			Er-doped ZBLAN	3489	–	28.91 M	1.38	40	54	
	BP	LPE	Dy-doped ZBLAN	3040	740 n–1.8 μ	47–86 k	0.5–1.0 μ	24–87	–	[306]
MXenes	Ti${}_{3}$C${}_{2}$T${}_{x}$	AAE	Nd:YAG	1064	359–688 n	109–186 k	0.66 μ	94.8	–	[324]
	Ti${}_{3}$C${}_{2}$T${}_{x}$	AAE	Yb	1065.89	480 p	18.96 M	0.47	9	56	[316]
	Ti${}_{3}$C${}_{2}$T${}_{x}$	AAE	Er	1555.01	159 f	7.28 M	410 p	3	∼62	
	Ti${}_{3}$C${}_{2}$T${}_{x}$	LPE	Er	1573.2	1.73 p	11.1 M	–	–	62.2	[373]
	Ti${}_{3}$C${}_{2}$T${}_{x}$	AAE	Tm	1976	2.4 μ	59 k	–	–	52	[331]
	Ti${}_{3}$C${}_{2}$T${}_{x}$	AAE	Er-doped ZBLAN	2786.2	1.04–28 μ	32.47–78.12 k	6.52–13.93 μ	210–1090	42.1	[336]
	Ti${}_{2}$CT${}_{x}$	AAE	Yb	1037.8	792 p	16.5 M	0.72	6.57	75	[326]
	Ti${}_{2}$CT${}_{x}$	AAE	Yb	1565.4	164 p	8.25 M	–	–	62	[56]
	Ti${}_{2}$CT${}_{x}$	AAE, LPE	Tm:LuAG	2027	178 n	1 k	189 μ	189	–	[333]
	Ti${}_{2}$CT${}_{x}$	AAE	Ho:YLF	2062.2	837 n	35.5 k	20.8 μ	341	–	[374]
	Ti${}_{2}$CT${}_{x}$	AAE	Er-doped ZBLAN	2798	1.83 μ–730 n	44.1–99.5 k	0.35–0.81 μ	16–80	33.1	[56]
	V${}_{2}$CT${}_{x}$	AAE	Yb	1064	72 f	38.5 M	1	38.5	71	[327]
	Ti${}_{3}$C${}_{2}$	AAE	Tm-Ho	1964	–	–	–	–	56	[330]
	Ti${}_{3}$CNT${}_{x}$	AAE	Er	1557	660 f	15.4 M	3.2 p	0.05	60	[315]
	Nb${}_{2}$C	MSD	Yb	1031.5	271 p	14.8 M	0.89	13.2	62	[375]
	α-Mo${}_{2}$C	CVD	Yb	1061.8	418 p	3.22 M	–	–	64	[376]
			Er	1602.6	1.81 p	1.88 M			45	
Hetero structures	Graphene/WS${}_{2}$	CVD	Nd:YVO${}_{4}$	1064	66–149 n	3.528–7.777 M	33.1	275	–	[343]
	Graphene/Bi${}_{2}$Te${}_{3}$	CVD	Yb:KYW	1037.2	236 f	41.84 M	19	550	65	[377]
	BP/Ti${}_{3}$C${}_{2}$	–	Er	1559.8	735 f	11.7 M	–	–	75	[350]
	Graphene/Bi${}_{2}$Te${}_{3}$	CVD	Er	1568.07	837 f	17.3 M	0.178	3.07	60.7	[340]
	MoS${}_{2}$/Graphene	Modified Hummers	Er	1567.2	9.31–19.12 μ	6.312–21.9 k	73.5–98.6	0.464–2.16	–	[341]
	Graphene/BP	LPE	Er	1529	865 f	295.6 M	–	–	48	[342]
	WS${}_{2}$/MoS${}_{2}$/WS${}_{2}$	MSD	Er	1562.66	296 f	36.46 M	0.686	25	90.3	[147]
	Bi${}_{2}$Te${}_{3}$/Graphene	Solvothermal synthesis	Nd:YAG	1980	238 n–1.22 μ	46–108 k	21.7 μ	2340	–	[344]
MoS${}_{2}$/Graphene	HI/E	Er:YSGG	2797	355 n	126 k	0.889 μ	112	–	–	[346]

*λ*, central wavelength; *τ*, pulse width; *f_rep_*, repetition rate; Power, average output power; Energy, single pulse energy; SNR, signal to noise ratio; CVD, chemical vapor deposition; MBE, molecular beam epitaxy; LPE, liquid phase exfoliation; ME, mechanical exfoliation; PM, polyol method; MSD, magnetron sputtering deposition; PLD, pulsed laser deposition; HI/E, hydrothermal intercalation/exfoliation method; Yb, ytterbium; Nd, neodymium-doped; Er, erbium; Tm, thulium; Ho, holmium; ZBLAN, ZrF_4_-BaF_2_-LaF_3_-AlF_3_-NaF; Pr, praseodymium; Lu, lutetium; and Dy, dysprosium.

MXenes, mostly fabricated by AAE method, have been widely used as SAs within a waveband from 1 to 3 μm due to the broadband saturable absorption property [316,324,331,336]. However, more research in the farther mid-infrared region deserve to be explored [318]. As for lasers enabled by 2D material-based heterostructures, the majority of them work in telecommunication bands, as well as using graphene and TMDs as SAs. Heterostructures based on other 2D materials, such as MXenes, are also worth investigating at higher operating wavelengths in lasers.

## 4. Perspectives

The unique optical properties of the newly developed 2D materials overcome the performance defects of traditional SAs and have been widely applied in various ultrafast lasers. The performance of SAs, such as the modulation depth and response time, has a crucial impact on the generation of ultrashort and high-energy pulses. Therefore, it is worth exploring SAs used in ultrafast lasers with excellent properties. In the early stage, NPR and NOLM were commonly used as artificial SAs. However, the instability of NPR makes it easy to be affected by the environment and hard to realize self-starting operations. NOLM possesses a low damage threshold that hinders the applications in ultrafast lasers with high power and a narrow wavelength range [43].

The emergence of low dimensional nanomaterials, including 2D and 3D materials, offers reliable options for SAs. 3D materials, such as Dirac semimetal Cd${}_{3}$As${}_{2}$, metal nanosphere, and nanoscale charcoal powder, have been investigated in the past few years [29,30,31,32,33,34,35,36]. Cd${}_{3}$As${}_{2}$ showed significant saturable absorption properties in the near- and mid-infrared regions [31]. Metal nanospheres have the advantages of wideband adjustability and an ultrafast response time [29]. In addition, nanoscale charcoal powder has a higher modulation depth of 26% and transmittance of 0.91 that benefits from the decrease of size [36].

2D and 3D materials both entail superiorities as SAs, such as excellent saturable absorption characteristics, ultrafast response times, and rich preparation methods. Compared with 3D SAs, 2D materials are worth investigating owing to their unique advantages. First, the controllability of the atomic layer thickness and bandgap structure of 2D materials provide a favorable condition for the adjustment of the physical and chemical properties [15]. Then, the insertion loss caused by the thin thickness of 2D materials is also lower than that of 3D materials in a laser cavity [37]. Moreover, a larger family of 2D materials can serve as SAs in the field of ultrafast photonics, which provide complementary properties among each other.

The performance of SAs can be enhanced by stacking van der Waals heterostructures made of different 2D materials [13,339]. Furthermore, the carrier migration and heat diffusion of 2D materials are confined in plane, making them possess specific optical and electrical properties, such as quantum Hall effect and ultrahigh carrier mobility [378,379,380]. However, the study of 2D materials started relatively late, and there are still many potential areas to be further explored. Here, the future developments of 2D materials in the field of ultrafast photonics are presented.

Exploiting high-performance SA materials: On the one hand, it is necessary to further discover new 2D materials with high performance, such as a wide operating band, high damage threshold, and fast relaxation time. On the other hand, we can adjust the structural parameters of existing 2D materials by means of surface modification, doping, and intercalation, so as to improve the properties of 2D materials and make them desirable SAs [63]. Taking graphene as an example, we can use polyethyleneimine for surface treatment to improve its wettability and optical properties, and the bandgap of graphene can be controlled by doping oxygen atoms. Furthermore, intercalating with bromine can maintain the high light transmittance and electrical conductivity of graphene [381,382,383].

Extending the broadband response range: Owing to the broadband absorption properties of 2D materials, they have an optical response from the visible to infrared bands. Therefore, the ultrafast laser can further extend the operating wavelength range. At present, ultrafast fiber lasers based on 2D materials have been widely used from the 1- to 3-μm bands, while the research on the mid-IR band beyond 3.5 μm is still in the early stages [63,190]. By further optimizing the laser design and selecting the ideal gain medium and SA, a wider range of ultrafast laser pulses can be obtained.

Improving the preparation methods: Bottom-up growth and top-down exfoliation methods can be used to prepare 2D materials; however, they both have disadvantages. Cost-effective microwave and LPE technologies are difficult to ensure high crystallinity and the quality of materials, and ME—which can produce high quality products—is complex and difficult to operate. Moreover, the consistency and reproducibility of 2D material samples still struggle to meet the requirements. In order to transform laboratory-scale production into industrial large-scale production, it is necessary to strengthen preparation theory research and explore the growth mechanism.

At present, CVD is a promising method due to its large size, good uniformity, and controllable number of layers [25]. However, due to the use of a variety of precursors and the complex growth process, there are still many problems in maintaining the controllability of 2D film preparations. Thus, it makes sense to further study the key parameters affecting the growth process of CVD, which can realize the quantity production of 2D materials with high quality and productivity.

Combining advantages through heterostructure: Although the current 2D materials used for SA have unique advantages, the limitations of a single material impede their potential as excellent SAs. The low saturation intensity of graphene has a positive effect on the SA, yet the 2.3% single-layer modulation depth places restrictions on its applications [384]. TIs have an ultrafast recovery time; however, the mode-locking stability and continuity are lower than that of graphene [70]. TMDs possess a large modulation depth and high damage threshold; however, the bandgap is too wide for pulse operation in the mid-IR band [385]. The BP-based ultrafast laser can be extended to the 3.5-μm band; nevertheless, BP is very unstable and easily oxidized in the air [49].

Despite the broadband optical response and strong absorption coefficient of MXenes, the limited preparation methods hinder their development and applications [316]. Therefore, combining the optical advantages of two or more 2D materials has become a new development direction of SA, which can effectively avoid the limitations of single material applications. At present, the commonly used method is stacking 2D materials by heterostructure, which can improve the performance of the materials based on theoptical complementary effect. In this way, one can obtain SAs with a larger modulation depth, wider absorption bandwidth, higher peak power, and better stability [345,386,387].

Exploring other ultrafast laser applications: In addition to saturable absorption, 2D materials have distinct optical and electrical anisotropy, which indicates their potential in light modulation and polarization detection. For example, in 2020, Sakakura et al. found that laser writing in quartz glass can produce geometric phase shifts [388], and thus the study of new characteristics, such as the polarization properties of 2D materials, could broaden their applications in ultrafast lasers.

## 5. Conclusions

In this review, we introduced the principles, preparation processes, characteristics, and applications of ME, solution-processed methods, deposition methods, MBE, and other widely used methods. Then, we made conclusions regarding the applications of commonly used 2D materials as SAs from the 1- to 3-μm wavelengths and further discussed the state of the art and challenges. Furthermore, an outlook for optimizing preparation methods and broadening the applications of 2D materials was also presented in the section on perspectives. Graphene can be prepared by multiple methods, including ME, CVD, and LPE. ME is often used in laboratory research due to its simple operation and high material integrity, while industrial large-area graphene can be prepared by LPE and CVD.

However, the ultrasonic process of LPE may lead to structural defects [389]. Thus, it is necessary to optimize the conditions of the ultrasonic process, such as the time, temperature, and reaction space [390]. CVD can achieve high quality and fast growth of graphene under a suitable pressure, temperature, and substrate, which is one of the main research directions. For example, graphene can be rapidly prepared by a static atmospheric pressure CVD system based on molecular thermal motion and a Cu substrate [391]. High-quality TIs can be obtained by ME with low cost; however, this has the drawbacks of low yield and difficulty in controlling the thickness [66,392].

In order to obtain high-purity TIs with uniform thickness, MBE can be used despite the need for vacuum environment and long preparation time [162,393,394]. LPE and the hydrothermal method can obtain high-quality TIs with simple processes, in which the suitable doping of impurities has important impacts on the characteristics of the TIs [88,89,395,396]. Linear, banded, and flake TIs can be prepared by the CVD method, whereas the growth rate is still a problem to be solved [143,396,397]. MBE is the earliest developed method to synthesize TMDs, and is mainly used in scientific experiments due to the high cost and low speed [28,398].

Later, the exfoliation of high-purity natural or synthetic bulk crystals by ME accelerated the research of TMDs [65,67,398,399]. However, ME lacks repeatability, and the products are not homogeneous. Therefore, CVD methods for large-scale preparation of TMDs were explored [146]. Recently, LPE and ME have been the two main methods for preparing BP. LPE is simple and effective but the layers of prepared BP are uncontrollable, which hinders the subsequent research on the optical properties of BP [82]. The ME method can obtain few or even single layer BP, while the yield and quality of BP remain to be improved. As an option, viscoelastic poly-dimethylsiloxane (PDMS) can be used for exfoliation rather than utilizing tapes [312].

Furthermore, BP is unstable, and the optical properties can be affected when exposed to air; thus, exploring proper methods for better storage is worthy of study. For example, we can use polymethyl methacrylate (PMMA) for surface encapsulation to inhibit the oxidation of BP material [400]. As a kind of novel 2D material, MXenes can be prepared by the AAE method [116,122,123], in which HF was used at first. Later, in view of safety and efficiency, HCl-LiF and fluorides, such as NH${}_{4}$HF, gradually became substitutes [123,124,125,126].

In addition, CVD can be used to prepare large-area MXenes without external functional groups, which are mostly used in the research of electronics and optics [138,401]. However, considering that CVD requires an ultrahigh reaction temperature, plasma-enhanced pulsed-laser deposition (PEPLD) is another adoptable method to decrease the preparation temperature and synthesize high-quality MXenes [402].

Regarding the performance of ultrafast pulses, the pulse energy and output power can reach up to the order of μJ and Watts, respectively [231,336,344]. In 2D material-based lasers, ultrashort pulses with a femtosecond width are preferred to be obtained by mode-locked operation in 1.5 μm. Most of Q-switched lasers using 2D materials as SAs generate pulses with pulse width from nanosecond to picosecond. Concretely, graphene and BP are mostly used in solid-state lasers that are commonly applied in the 1-μm region. TMDs and TIs both tend to generate Q-switched pulses with high pulse energy and output power, which are beneficial to the applications in high power lasers.

MXenes and heterostructures are desirable alternatives to generate pulses with high SNR, indicating high stability. In terms of broadband operations, graphene, BP, and MXenes have priorities due to broadband saturable absorption properties; however, the easy oxidation of BP limits its practical applications. In addition, graphene, TIs, and MXenes show innate talent for high pulse energy while there is still room for TIs to enhance the mode-locked stability. As for BP and TMDs, SAs with better properties and stability as well as convenient and low-cost fabrication methods are worth exploring in order to take advantages of their full potential in ultrafast lasers.

2D materials are popular in applications of ultrafast photonics, such as detection, luminescence, and modulation, due to the outstanding photoelectric properties. Ultrafast photonics based on 2D materials have become a highly active field of research. Since graphene was applied in ultrafast lasers, people have concentrated an enormous amount of effort on the characteristics of 2D materials, in order to develop the excellent performance of ultrafast photonic devices. Although some difficulties and challenges inhibit the large-scale and high-quality preparation of 2D materials, the broad development prospect serves as an irresistible trend of the times. We believe that, with the in-depth study of the properties and preparation processes, ultrafast photonics based on 2D materials will gradually develop from scientific research to industrial technology applications and, thus, bring innovation to fields such as optics, electronics, and biology.

## Data Availability

Not applicable.
